# Implementation of electronic signposting to interventions that prevent cancer: A realist review

**DOI:** 10.1371/journal.pdig.0001770

**Published:** 2026-10-05

**Authors:** Tracey J. Brown, Natalie An Qi Tham, Felix Naughton, Henry Goodfellow, Louise Hull, Helena Jopling, Helen M. Parretti, Alex T. Ramsey, Adam P. Wagner, Zarnie Khadjesari

**Affiliations:** 1 School of Health Sciences, University of East Anglia, Norwich, England; 2 Primary Care and Population Health, University College London, London, England; 3 Leicester Diabetes Centre, The University of Leicester, Leicester, England; 4 Department of Public Health, West Suffolk NHS Foundation Trust, Bury St. Edmunds, England; 5 Norwich Medical School, University of East Anglia, Norwich, England; 6 Department of Psychiatry, Washington University School of Medicine, St. Louis, Missouri, United States of America; 7 National Institute of Health Research (NIHR) Applied Research Collaboration (ARC) East of England (EoE), Cambridge, England; McGill University Faculty of Medicine and Health Sciences, CANADA

## Abstract

Uptake of evidence-based interventions that prevent cancer is very low. Electronic signposting using digital messages sent via electronic healthcare systems is increasingly utilised to improve service reach and uptake. However, theory for optimising the implementation of electronic signposting to cancer prevention interventions is lacking. We sought to generate a novel programme theory to illuminate what type of electronic signposting works, for whom and in what circumstances. A realist review, informed by Realist And Meta-narrative Evidence Syntheses: Evolving Standards (RAMESES I), was conducted. Medline, EMBASE, CINAHL, Scopus, PsycINFO, ERIC and AMED databases, and grey literature were searched. Studies that contributed information on context and mechanisms of action for electronic signposting to cancer prevention interventions for adults in any healthcare setting were included. Studies were assessed for quality based on relevance, richness and rigour. Realist synthesis and input from stakeholders, including patients, was used to aid development of our novel programme theory. Thirty studies were included, 26 of which were conducted in the United States. 57 individual context-mechanism-outcome configurations were identified. For patients, memorable messages using wording tailored to patient characteristics, and use of a popular communication channel (e.g., SMS text) improved ‘buy-in’ and usability. For providers, a good fit with organisational priorities, workflow, and establishing compatibility with existing technical systems was important. Increasing optimism of technology and ensuring electronic signposting did not risk existing patient-provider relationships were also key to success. To reduce cancer and cancer disparities, policymakers and providers need to implement electronic signposting in ways that promote ‘buy-in’, ensure perceived usability, and nurture positive attitudes towards technology. The field would benefit from further qualitative insight, health economic analysis, exploration of equity and long-term outcomes, and research in non-USA contexts. Protocol registration: PROSPERO [CRD42024586907].

## Author summary

Cancer is responsible for growing health and economic problems worldwide. Effective cancer prevention interventions exist, but their uptake can be very poor. Electronic signposting uses electronic health records to identify individuals at risk and signpost these individuals to health services using digital messages, such as text messages. This could be a low-cost and effective way to increase uptake and use of cancer prevention interventions, but knowledge for how best to use electronic signposting is low. We aimed to understand what type of electronic signposting works, for whom, and in what circumstances. We found that simple memorable messages, customised to different types of patients, increased use of messages that were signposting patients to cancer prevention interventions. For health professionals, ensuring a good fit with organisational priorities and systems, increased the chances of sending messages. It was also important to maintain and build on existing patient-provider relationships. Our theory shows that electronic signposting needs design input from a range of patients and professionals to be most effective.

## Introduction

Evidence indicates that interventions which prevent cancer are effective, such as those supporting smoking cessation, weight management, alcohol reduction, physical activity, cancer screening and vaccination programmes [1,2]. However, lack of knowledge of available cancer prevention services is a major barrier to their uptake, driven in part by low levels of routine referral to cancer prevention services [3]. For example, use of smoking cessation services is as low as 0.2% (of smokers) in Europe [4], and as few as 5% of patients are screened for alcohol consumption [5,6]. There are also marked cancer health inequalities, with lower socio-economic groups being less likely to access interventions and healthcare services [7]. Effective strategies are needed to help overcome barriers and harness enablers to intervention uptake to prevent cancer incidence and reduce cancer disparities.

The use of electronic health records is now widespread across healthcare systems and has the potential to identify large numbers of patients at higher risk of cancers [8]. Electronic signposting (eSignposting) is the proactive referral of these patients to interventions using digital messages. It is thought to be a low cost strategy to improve patient engagement and access to cancer prevention services [8,9]. We define eSignposting as: using electronic health records to systematically identify at risk individuals and to signpost them to services or interventions using digital forms of communication (SMS text, emails, patient portal messages, automated telephone calls). eSignposting has the potential to act as a powerful adjunctive intervention; defined as a change method to increase recipient initiation or engagement with interventions [10]; to increase the uptake and use of cancer prevention interventions. eSignposting has successfully improved the reach and engagement of cancer prevention strategies such as vaccinations, cancer screening, and behaviour change interventions for smoking cessation, weight management and alcohol reduction [11–14]. For example, text messages to patients identified via electronic health records increased colorectal cancer screening rates by 16.5% [14]. Despite the effectiveness of eSignposting, there is limited understanding of how best to implement this adjunctive intervention. Many implementation failures are reported for eHealth interventions which is detrimental to healthcare and often costly [15–17]. Failure may arise through a variety of factors such as human error, lack of training and weaknesses in infrastructure and cybersecurity [18]. eSignposting operates within complex stratified systems and requires deep understanding of how and why it may or may not ‘work’ (is it effective, feasible, acceptable?) in real world settings. Lewis et al [19] underline the importance in understanding mechanisms and causal pathways to maximise the implementation and effectiveness of interventions. Realist reviews aim to uncover these causal pathways [20]. Realist reviews of the implementation of healthcare technological interventions have highlighted the importance in understanding contextual factors and applying theoretical knowledge to ensure effective implementation [17,21,22]. A greater insight into how to implement eSignposting is crucial to increase uptake, effectiveness and sustainability of this relatively novel adjunctive intervention within healthcare settings. Optimising the implementation of eSignposting will benefit health professionals by aiding care provision and in turn, will benefit patients by improving access to effective care.

Using realist review methodology [20], we aimed to develop a novel programme theory to illuminate the implementation of electronic signposting to interventions that prevent cancer in generally healthy adults. We aimed to answer the following research questions: what type of electronic signposting works, for whom, and in what circumstances? Our realist review will generate the first programme theory of eSignposting to cancer prevention interventions.

## Materials and methods

A realist review approach is used to help explain how and why an intervention works in addition to understanding intervention effectiveness, and is increasingly recognised as a valuable approach to generating new theories to comprehend complexities of healthcare interventions [20,23]. Realist methodology was informed by Realist And Meta-narrative Evidence Syntheses: Evolving Standards (RAMESES I) guidance [24] and we followed the RAMESES reporting standards [25,26]. The RAMESES checklist is shown in S1 Table. The methodological steps outlined by Pawson et al [20] were used to: 1) scope the literature to develop an initial programme theory; 2) search for relevant articles; 3) appraise studies and extract data; and 4) synthesise the evidence to produce a refined programme theory. Our review protocol is registered on PROSPERO (CRD42024586907).

### Development of initial programme theory

Informal literature scoping in Medline and Google Scholar was used to define the scope of the review and to develop initial programme theories. A tentative set of theories were developed using the structure of ‘if, then’ statements to represent causal pathways considered likely based on broad scoping, and by drawing on concepts of the NASSS Framework which theorises ‘Nonadoption, Abandonment, Scale-up, Spread and Sustainability’ of healthcare technologies [16]. This framework was selected due to its focus on sustaining implementation of technological health interventions. ‘If, then’ statements were presented to professional stakeholder panel members in meetings or via email. Panel members consisted of two general practitioners, a health economist, a psychologist, a public health officer, and a consultant in public health medicine. Views of patient and public involvement (PPI) representatives were also included by discussing face-to-face with them how and why eSignposting might work. Stakeholder input is a common and valued element of developing robust programme theories [27]. Based on stakeholder feedback, which was returned orally or by email, the broad theoretical areas were subdivided into explicit context-mechanism-outcome (CMO) configurations to enable detailed understanding of causal pathways. CMO configurations offer explanations of how a particular context activates a particular mechanism (an underlying reason) to generate an intervention or implementation outcome [20]. Evidence was subsequently sought to test and refine these CMO configurations.

### Searches

Searches were conducted on 27^th^ September 2024, in AMED, CINAHL, Embase, ERIC, Medline, PsycINFO and Scopus using both database-specific subject headings and free-text keywords. Search terms focused on: electronic health records; electronic communications (telephone, email, patient portal); and cancer prevention interventions. We also searched the grey literature (CORE, Google Scholar, Grey Matters, International HTA database, Proquest, and key cancer charity, health and digital websites). No date criteria or other limitations were applied to searches. Search strategies are shown in S2 Table. Reviews which were highly relevant to the research question were hand searched for relevant articles, and forward and backward citation searching of included studies was used to seek further eligible articles.

### Study inclusion and selection

Studies of generally healthy adults (without illness, excepting chronic illnesses commonly linked to primary cancer risk, e.g., diabetes) which could contribute information on context and mechanisms of action for electronic signposting to interventions that prevent cancer in any healthcare or community setting were included. Eligible cancer prevention interventions were defined a priori as: smoking cessation; weight management; alcohol reduction; increased physical activity; cancer screening; cancer prevention vaccination; or sun safety [1,2]. There was no specific comparison group since we sought an understanding of different contexts, mechanisms and outcomes. Outcomes included any measures of behaviour change, health and wellbeing, implementation, and service measures. Studies not focused on electronically signposting individuals to cancer prevention interventions were excluded.

Studies were screened for eligibility by title and abstract in Rayyan (Rayyan.ai). Screening was conducted by the first author (TJB) and independently duplicated by the second author (NAQT) at 40%. TJB and NAQT met to compare decisions, and differences were discussed to enable refinement of screening criteria. Full texts were assessed for inclusion based on ability of the text to contribute meaningfully to programme theory building, in line with quality appraisal. Full text inclusion was independently duplicated at 20% (TJB and NAQT), with any discrepancies resolved by discussion to ensure systematic application of inclusion criteria.

### Study quality assessment

Quality appraisal was based on principles outlined by Dada et al [28]. Quality appraisal in realist reviews considers how much information is present on CMOs, and if the data is considered trustworthy and coherent assessed in terms of relevance, richness and rigour [29]. *Relevance,* which refers to whether an article can contribute to theory building [28, 29], was the primary criteria for initial quality appraisal. *Richness*, which refers to the degree by which articles can be used to develop or test CMOs [28,29], was subsequently assessed, resulting in the exclusion of studies which we judged were conceptually ‘thinner’ and lacking sufficient depth to contribute meaningfully to the development or testing of CMOs. We did not exclude studies based on assessment of their *rigour*, but since *rigour* was relevant to our interpretation of the findings, studies were classified into high, medium, or low quality based on their trustworthiness and coherence as outlined by Dada et al [28]. Quality assessment was independently duplicated at 20% (TJB and NAQT) and differences resolved through discussion and consensus of quality criteria.

### Data extraction

The following data considered pertinent to the research question were extracted into bespoke Excel tables: study type; setting; theoretical underpinning; patient characteristics; eSignposting characteristics (frequency, mode and content); intervention and implementation strategies; intervention and implementation outcomes. Articles were imported into NVivo to extract contexts, mechanisms and outcomes. Data extraction was both deductive using codes informed by the initial programme theory, and inductive to illuminate any additional CMOs. Authors of included articles were emailed to seek any additional information relating to their manuscripts. A 20% sample of studies was extracted independently (TJB and NAQT), with discrepancies resolved by discussion to increase reliability of data extraction. Data extraction continued until saturation, when included articles did not contribute new causal mechanisms.

### Realist synthesis

Analysis and synthesis were conducted by the first author in consultation with the research team. Views of stakeholders were re-sought following data synthesis to check validity of findings. Codes from NVivo were used to categorise and organise findings. Studies were compared and contrasted, to determine recurrent patterns. Data which confirmed, refuted or refined the programme theory were recorded against each initial CMO configuration, and new CMOs were incorporated and listed as programme theories. Causal mechanisms were conceived using retroduction, the ‘inference to theorize and test hidden mechanisms’ [30]. Based on theoretical underpinning of our included studies and relevance to mechanisms for eSignposting, we also drew on the NASSS [16], MINDSPACE [31], and COM-B [32] established frameworks to refine each CMO configuration. During data synthesis we moved back-and-forth between these frameworks and our CMO configurations to aid hypothesis of how and why eSignposting might work best.

Whilst the methodological steps above appear separate, in reality the process was iterative [25,26]. As the review progressed, we decided to exclude studies that focused on individuals aged under 18 years, as we hypothesised that mechanisms applying to children, or using parent proxies, may operate differently to those of adults. We had expected to run additional searches, but reached data saturation after initial searches, thus further searches were not conducted. Given the relatively large dataset, we started by extracting the studies considered richest, i.e., having a relatively high number, or rich descriptions of CMOs. Once data saturation was reached, remaining studies considered as having a lower number, or less rich descriptions of CMOs, were excluded based on the criteria of insufficient richness.

## Results

### Included studies

From 3,532 unique articles identified, 305 articles were selected for full text assessment, with the final inclusion of 49 articles relating to 30 studies [12–14, 33–59] in our realist synthesis. We judged studies to be of high (n = 27) or medium (n = 3) relevance; to be of high (n = 11) or medium (n = 19) richness; and to be of high (n = 25) or medium (n = 5) rigour. Ten authors [12,14,37–39,41,42,51,54,59] responded with additional information on their studies. The flowchart of study identification and screening is shown in Fig 1.

**Fig 1:**
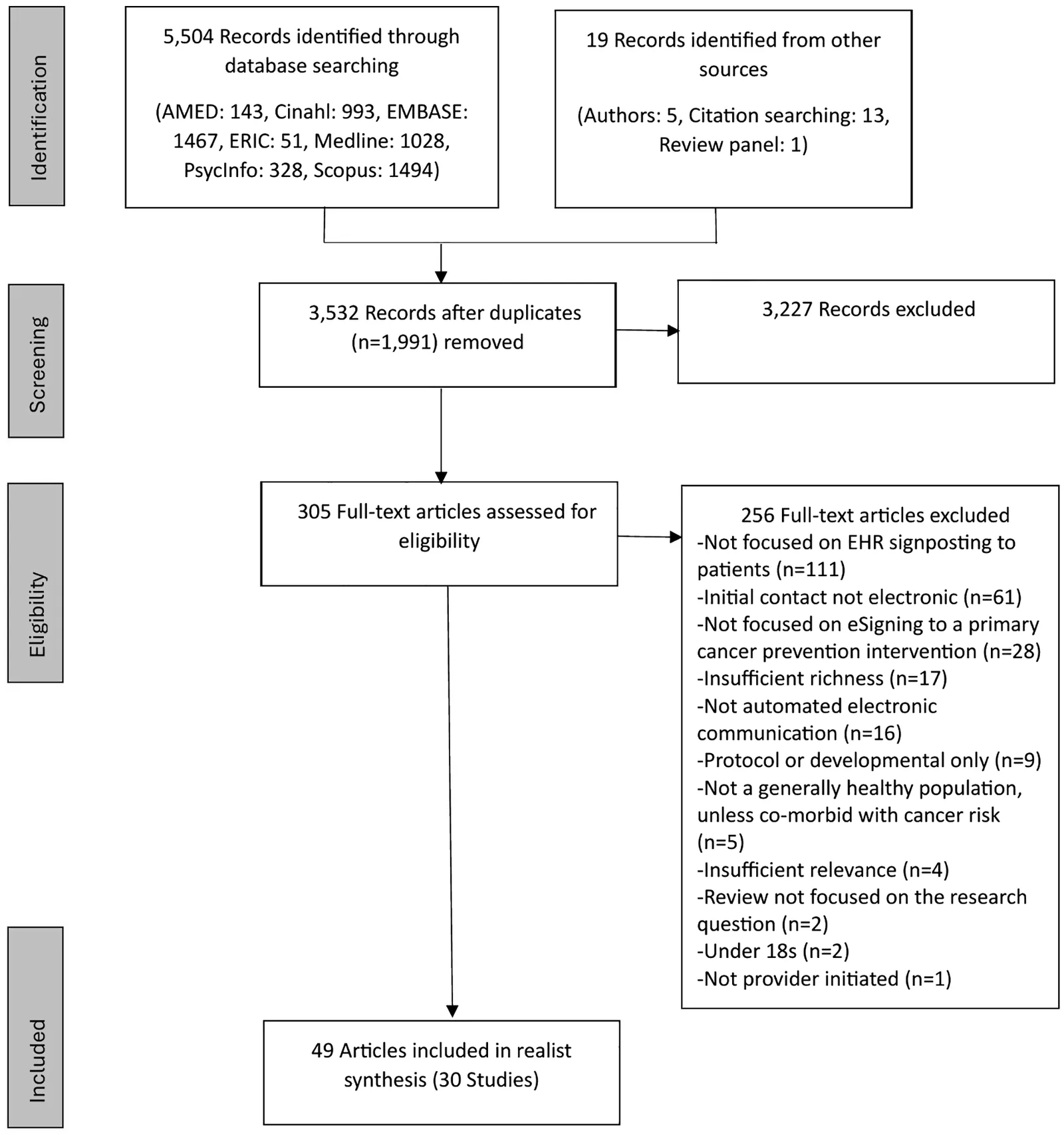
Flow diagram.

### Study characteristics

Studies were described as: randomised (n = 12); observational (n = 5); single-arm (n = 3); cross-sectional (n = 3); reviews (n = 2); quality improvement (n = 2); correlational (n = 1); mixed methods implementation (n = 1); or qualitative (n = 1) in design. Most studies were conducted in the USA (n = 26), with remaining studies conducted in the United Kingdom (n = 2), Australia (n = 1), and Saudi Arabia (n = 1). Cancer prevention interventions addressed: colorectal cancer screening (n = 7); smoking cessation (n = 5); breast cancer screening (n = 3); lung cancer screening (n = 3); physical activity and nutrition (n = 3); weight management (n = 2); alcohol reduction (n = 1); alcohol reduction and smoking cessation (n = 1); self-care behavioural intentions (n = 1); cervical screening (n = 1); chronic disease management and prevention (n = 1); HPV vaccination (n = 1); and wellness visits and cervical screening (n = 1). Settings were: primary care or general community practices (n = 13); health systems (n = 5); academic medical centres (n = 3); hospitals (n = 3); community settings (n = 2); digital health organisations (n = 1); general healthcare settings (n = 1); military healthcare systems (n = 1); and tribal healthcare systems (n = 1). Summary characteristics of included studies are reported in Table 1, with full characteristics in S3 Table.

**Table 1:** Characteristics of included studies.

Author and year	Quality appraisal	Cancer intervention	Study type, Setting	eSignposting characteristics	CMOCs identified
Ackermann 2023 [34]	Relevance: High Richness: High Rigour: Medium	Weight-management	Randomised feasibility trial USA: 1 general internal medicine practice	1 EHR portal message offering services to support weight-loss. Followed by ‘periodic’ messages encouraging use of resources.	1, 5, 7, 8, 12, 16, 17, 20, 21, 23, 24, 29, 32, 36, 37, 38, 41, 42, 45, 46, 47, 48, 50, 51, 52, 54, 56
Azevedo 2023 [35]	Relevance: High Richness: Medium Rigour: High	Self-care behavioural intentions	Correlational USA: Community setting	Participants were presented with hypothetical scenarios that contained six patient portal messages.	1, 2, 6, 7, 22, 24, 30, 46, 47, 53
Bayard 2022 [36]	Relevance: Medium Richness: Medium Rigour: Medium	Breast cancer	Review USA: General healthcare	Review concerning EHR notifications for breast cancer.	4, 6, 8, 16, 20, 21, 32, 44, 46, 49
Begnaud 2017 [37]	Relevance: High Richness: Medium Rigour: High	Lung cancer screening	Randomised feasibility trial USA: 1 academic medical centre	1 portal message informing patients of a new preventive care service, with a duplicate message sent after 30 days to patients who failed to read the initial message. Patients were sent a follow-up portal message indicting eligibility for lung cancer screening. Control group received no messages.	1, 6, 8, 17, 20, 21, 26, 29, 45, 48, 57
Combest 2019 [13]	Relevance: High Richness: Medium Rigour: Medium	Exercise and nutrition	Single-arm pragmatic trial USA: Military healthcare system	1 health promotion email including follow-up information for appointments; followed by a survey for users who consented to this.	1,12, 13, 17, 24, 34, 41, 45, 47, 51, 54, 55
**Coronado 2023 PROMPT study** [38] Coronado 2019 [60] Coronado 2021 [61] Coronado 2024 [62] Escaron 2022 [63] Schneider 2023 [64] Thompson 2019 [65]	Relevance: High Richness: High Rigour: High	Colorectal cancer screening	Stepped-wedge, cluster randomised trial USA: Primary care clinics	Single text message 1–2 days before receipt of mailed FIT kit encouraging test completion. The phone call primer group received 1 live phone call prior to postage. The control group received only reminder phone calls (no primers).	2, 4, 5, 6, 8, 10, 11, 17, 20, 21, 23, 24, 26, 30, 31, 32, 33, 36, 41, 45, 46, 47, 48, 49, 51, 52, 54, 55
**Dharod 2019** [39] Patel 2020 [66]	Relevance: High Richness: High Rigour: High	Lung cancer screening	Single-arm pragmatic trial USA: 1 large academic health system	Single patient portal invitation to visit a lung cancer screening interactive website.	1, 5, 6, 14, 16, 17, 20, 21, 22, 26, 28, 29, 34, 38, 39, 41, 42, 45, 46, 47, 50, 52, 55, 56
**Eden 2020** [40] Eden 2015 [67]	Relevance: High Richness: Medium Rigour: High	Breast cancer screening	Cross-sectional pilot study USA: General internal medicine clinic at an academic medical centre	Patient portal invitation to use a breast cancer decisional screening tool, with reminders sent at 2 weeks, and 1 month for non-completers.	1, 5, 6, 20, 22, 24, 26, 41, 43, 46, 47, 52, 55
El-Osta 2021 [41]	Relevance: High Richness: High Rigour: High	Smoking cessation	Prospective cohort UK: General practices	SMS or email message linking to an online portal enabling patients to request pharmacological support. For patients making medication requests, further messages were sent to enable repeat prescription requests every 4 weeks.	1, 2, 5, 8, 10, 11, 12, 14, 17, 18, 21, 24, 34, 36, 38, 39, 41, 44, 45, 46, 47, 52, 54, 56
Erdmann 2022 [42]	Relevance: High Richness: Medium Rigour: High	Smoking cessation	Quality improvement randomised clinical trial USA: 1 large health system	1 portal message encouraging a smoking quit attempt. Patients in the survey groups who responded, were sent a care plan via portal message. 4 intervention groups: message from physician or from health system with or without survey to asynchronous care.	1, 6, 7, 10, 11, 16, 17, 20, 21, 22, 25, 29, 35, 41, 45, 46, 54, 56
**Hess 2014** [43] Fischer 2013 [68] Rief 2017 [69]	Relevance: High Richness: Medium Rigour: High	Chronic disease management and prevention	Prospective cohort USA: Primary care practices within a single health system	Notifications delivered through a personalised health record, by email and portal. Participants with disease prevention gaps received up to 3 weekly messages regarding services due within 2 months. Messages repeated up to every 2 months for a new or continuing prevention gap. Messages encouraged use of disease prevention services.	5, 6, 10, 11, 12, 21, 22, 24, 26, 30, 35, 36, 41, 45, 47, 52, 55, 56
**Houston 2015 Quit-Primo study** [44] Sadasivam 2011 [70] Sadasivam 2013 [71]	Relevance: High Richness: High Rigour: High	Smoking cessation	Hybrid type 2 implementation randomised controlled trial USA: Community-based clinical practices	Email via ePortal with up to 10 reminders to register on a smokers quit website. Pushed varied motivational emails were also sent based on benefits, planning, and managing quitting. Messages were tailored to readiness to quit. In the first week, 4 motivational emails were sent followed by 2 emails per week. The control group received paper referral only.	1, 2, 5, 8, 12, 13, 16, 17, 19, 20, 21, 27, 29, 32, 34, 35, 36, 38, 39, 40, 42, 43, 45, 47, 48, 51, 52, 53, 55
Hwang 2023 [45]	Relevance: High Richness: Medium Rigour: Medium	Colorectal cancer screening	Cross-sectional pilot study USA: Community setting	Text message programme to inform CRC screening decision-making, with branching logic determining what and how much programme content was sent.	1, 5, 6, 7, 8, 21, 22, 24, 46, 47, 54, 55
Kearney 2024 [46]	Relevance: High Richness: Medium Rigour: High	Lung cancer screening	Pragmatic randomised trial USA: 1 large academic hospital	Portal questionnaire group used a single ‘helpfulness’ message. Text message groups used either ‘gain,’ ‘loss’ or ‘helpfulness’ framing and were sent a reminder text.	1, 4, 5, 12, 17, 20, 23, 24, 26, 28, 29, 46, 47, 52
**Khadjesari 2022** **OptiMine** [12] Blueprint [72]	Relevance: High Richness: High Rigour: High	Alcohol reduction; smoking cessation	Mixed methods implementation study UK: 1 NHS hospital	Text messages signing to NHS Smokefree, or Drink Free Days apps, with 1 reminder text.	1, 4, 6, 7, 8, 9, 10, 11, 12, 13, 14, 15, 16, 17, 20, 22, 23, 24, 25, 26, 27, 28, 30, 32, 33, 34, 35, 36, 38, 39, 40, 41, 42, 43, 44, 45, 46, 47, 48, 49, 51, 52, 53, 54, 55, 56, 57
Krebs 2020 [47]	Relevance: High Richness: Medium Rigour: High	Smoking cessation	Randomised trial USA: Urban hospitals	Initial call to action smoking quit text message sent with next day reminder; at 30 days, a second message with next day reminder was sent. Participants were randomised to one of six messaging sequences.	1, 4, 8, 11, 12, 17, 20, 26, 29, 45, 46, 49, 53, 55, 56
**Lafata 2022 e-assist colon health study** [48] Lafata 2019 [73] Tabriz 2019 [74]	Relevance: High Richness: High Rigour: High	Colorectal cancer screening	Randomised controlled trial USA: 1 large health system	Patient portal message to aid CRC screening decision making and motivate CRC screening, with follow-up module sent 2 weeks later. The control group received links to CRC screening information only.	1, 2, 4, 5, 10, 17, 20, 21, 22, 24, 26, 29, 32, 33, 34, 38, 45, 46, 51, 52, 53, 54, 55
Liang 2022 [33]	Relevance: High Richness: Medium Rigour: High	Wellness visits and cervical screening	Observational study USA: 1 large health system	Initially two emails: Email A focusing on curiosity, and Email B focusing on exclusivity. Email B was deemed most effective for roll-out. Multiple messages could be sent to encourage appointment booking. The same message principles were amended for portal reminders. The control group received only standard health maintenance reminders.	1, 2, 4, 10, 12, 13, 17, 20, 23, 26, 28, 29, 30, 31, 41, 46, 53
Lockwood 2023 [49]	Relevance: High Richness: Medium Rigour: High	Exercise and nutrition	Single-arm observational pilot study USA: Digital health organisation	Emails or text messages to recruit to a heart health digital intervention.	1, 4, 7, 8, 10, 17, 22, 26, 30, 34, 46, 47, 49, 57
Mahoney 2018 [50]	Relevance: High Richness: High Rigour: High	Smoking cessation	Cross-sectional USA: Urban and rural primary care medical offices in medically underserved locations	Automated voice recognition telephone calls to encourage smoking cessation. Calls were made over 6 months and attempted monthly, with re-attempts where ‘no answer’ was returned (after 3 unanswered calls, additional calls were deferred until the following month).	10, 11, 12, 13, 17, 20, 25, 26, 34, 35, 39, 41, 43, 45, 46, 47, 49, 52, 53, 54, 56, 57
**McIntosh 2024 SMARTscreen study** [14] McIntosh 2025 [75] Wood 2022 [76]	Relevance: High Richness: High Rigour: High	Colorectal cancer screening	Cluster randomised controlled trial Australia: General practices	A single text message sent 1 month before receiving the bowel screening kit. The control group received usual care of at-home screening without primer message.	1, 3, 4, 6, 7, 9, 10, 11, 14, 17, 21, 24, 25, 26, 29, 30, 32, 33, 39, 42, 43, 45, 46, 48, 49, 51, 52, 54
McVay 2022 [51]	Relevance: High Richness: High Rigour: High	Weight-management	Single-arm pilot trial USA: Family health clinics	Portal message before primary care appointment, followed by 8 follow-up messages over 12 weeks by patient portal, or email if not registered as a portal user to encourage weight-management.	1, 5, 6, 8, 10, 11, 12, 13, 17, 20, 21, 22, 23, 24, 29, 32, 33, 38, 41, 45, 46, 47, 52, 53, 54, 55
Mehta 2018 [52]	Relevance: High Richness: Medium Rigour: High	Colorectal cancer screening	Randomised controlled trial USA: General internal medicine clinics at an academic medical centre	Patient portal invitation to encourage ordering or use of a mailed FIT kit, with reminders sent to patients in the opt-in arm who did not respond within 3 weeks (no reminders were sent to opt-out patients).	1, 3, 5, 10, 17, 20, 21, 23, 24, 37, 46, 51, 53
Miller 2021 [53]	Relevance: High Richness: Medium Rigour: High	Diet and physical activity	Quality improvement study Saudi Arabia: Primary care clinic	Patient portal invitation to view lifestyle education videos sent weekly for 5 weeks, and a patient portal reminder to return to clinic within 3 months for a blood pressure assessment.	1, 4, 6, 12, 13, 17, 26, 29, 34, 35, 51, 54
Muller 2017 [54]	Relevance: High Richness: Medium Rigour: High	Colorectal cancer screening	Randomised controlled trial Alaska, USA: Tribal healthcare system	1 text message and up to 2 reminder text messages sent to encourage CRC screening. The control group received no text messages.	1, 4, 6, 8, 17, 20, 21, 26, 34, 35, 37, 39, 46, 47, 49, 52, 54
North 2021 [55]	Relevance: High Richness: Medium Rigour: High	Breast cancer screening	Observational cohort comparison USA: Primary care practices of a multi-speciality group practice	1 email/ text/ push notification inviting patients to self-schedule for mammography via website/ mobile app.	10, 11, 17, 20, 21, 26, 27, 29, 35, 45, 46
Peitzmeier 2016 [56]	Relevance: High Richness: Medium Rigour: High	Cervical screening	Randomised controlled trial USA: Urban community health centre	Email outreach; letter outreach; telephone outreach; or multi-modal outreach (email+letter+telephone) inviting cervical screening. The email groups received 3 outreach attempts. The control group received usual care with providers offering cervical screening as needed.	4, 6, 8, 12, 17, 20, 21, 23, 26, 34, 46, 54
Stephens 2019 [57]	Relevance: Medium Richness: Medium Rigour: Medium	HPV vaccination	Review USA: Health and community settings	Review concerning text, email and app reminders, EHR alerts (and social media).	2, 5, 8, 13, 16, 17, 20, 21, 24, 25, 26, 36, 37, 45, 46, 48, 56
Weaver 2015 [58]	Relevance: Medium Richness: Medium Rigour: High	Colorectal cancer screening	Qualitative USA: Community-based primary care practices	Qualitative study exploring patient attitudes towards CRC screening texts from providers, and desired content established using text message examples.	1, 3, 5, 6, 10, 20, 30, 31, 33, 45, 46, 47, 48, 49
**Yoon 2021 ATTAIN study** [59] Chevinsky 2021 [77] West 2023 [78]	Relevance: High Richness: High Rigour: High	Alcohol reduction	Quality improvement pilots USA: Preventive medicine, and primary care clinics	Portal messages, and email invitations to view messages, to screen for unhealthy drinking behaviours. Messages were sent 1 day, 3 days, 7 days, or 14 days prior to appointment date.	2, 4, 8, 11, 13, 14, 17, 20, 21, 22, 24, 25, 29, 34, 38, 42, 43, 45, 46, 51, 52, 56, 57

Abbreviations used: EHR (electronic health record); CRC (colorectal cancer); CMOCs (context-mechanism-outcome configurations); FIT (fecal immunochemical test).

Bold print highlights the main paper for studies which contained more than one article.

### eSignposting characteristics and outcomes

Most studies used portal notification(s) (sent via a patient portal platform, which integrates with patient health records), often in combination with email(s) or SMS text(s) (n = 18). Other included studies used SMS text(s) alone (n = 7), email(s) alone (n = 2), a combination of SMS text(s) and email(s) (n = 2), and one study used automated voice recognition (software combined with automated calling) via telephone (n = 1). In most studies more than one message was sent in the form of reminders, or follow-up information (n = 20), with six studies sending a single message, or additional content to responders only (n = 6), and remaining studies were reviews or based on hypothetical scenarios with no specific frequency reported (n = 4) (S3 Table).

In studies which reported a form of reach or engagement with electronic messages (n = 23), outcomes were predominantly considered to be successful. We defined reach as the percentage of patients reading the messages, and found reach ranged from 19% [56] to 86% [39]. We defined engagement with messages as an action beyond reading messages, e.g., click through rate. Engagement was measured by a variety of approaches limiting comparisons across studies. The lowest engagement was reported as 6.9% of patients responding to a portal questionnaire regarding smoking habits [46]. The highest rate of engagement was reported as 69% of patients viewing materials for a diet and lifestyle programme [53]. There was no discernible pattern linking reach or engagement with the mode, or number of eSignposting messages sent. In studies reporting a health behavioural outcome (n = 19), most perceived promising results in cancer prevention behaviours, although statistical significance was often lacking or not possible to calculate due to study design. Comparisons across studies reporting health behavioural outcomes were not possible due to the heterogeneous nature of included studies. However, we did not observe any pattern between behavioural outcomes and eSignposting mode or frequency. Most articles noted that electronic messaging offered potential for high patient engagement at relatively low cost.

Most studies (n = 17) reported a theoretical underpinning, or process of intervention development, whilst remaining studies reported no underpinning (n = 13). The most common theories applied were implementation frameworks [12,14,44,45,51,57], or behavioural economic theories [33,46,52]. Most studies (n = 20) employed implementation strategies which used: professional or leadership co-design alone [34,39,46,48,49,53,54]; patient co-design alone [43,47]; professional and patient co-design [12,50,51,59]; staff training alone [14]; pilot testing [33,37,40]; or a combination of co-design and training [38,42,44] to refine methodology (S3 Table). Due to the heterogeneous nature of studies, we found no clear link between implementation strategies and intervention effectiveness. Only one qualitative study was identified [58], which focused on patient insight. A further nine mixed-methods studies contributed additional qualitative insight, covering: both patient and provider views [12,14,34,38,40,51]; patient only views [43,47], or provider only views [44]. Whilst the proportion of qualitative data would ideally have been higher, these studies contributed balance and insight into both patient and provider perspectives, which facilitated refinement of our programme theory.

### Refined programme theory

A total of 57 CMO configurations were developed as shown in S4 Table with example excerpts. The CMO configurations were grouped into seven theoretical areas during initial programme theory development, and these theoretical areas were supported and retained following refinement of the programme theory. These theoretical areas were: 1) patient ‘buy-in’ to eSignposting (motivation to engage); 2) provider ‘buy-in’ to eSignposting (motivation to deliver); 3) patient perceived system usability (access and confidence with digital devices); 4) provider perceived system usability (access and confidence with digital infrastructure); 5) patient ‘techno-optimism’ (optimism in technology to receive eSignposting messages); 6) provider ‘techno-optimism’ (optimism in technology to deliver eSignposting messages); and 7) patient-professional solidarity (influence of eSignposting on patient-professional relationships). Our overarching programme theory is depicted in Fig 2.

**Fig 2:**
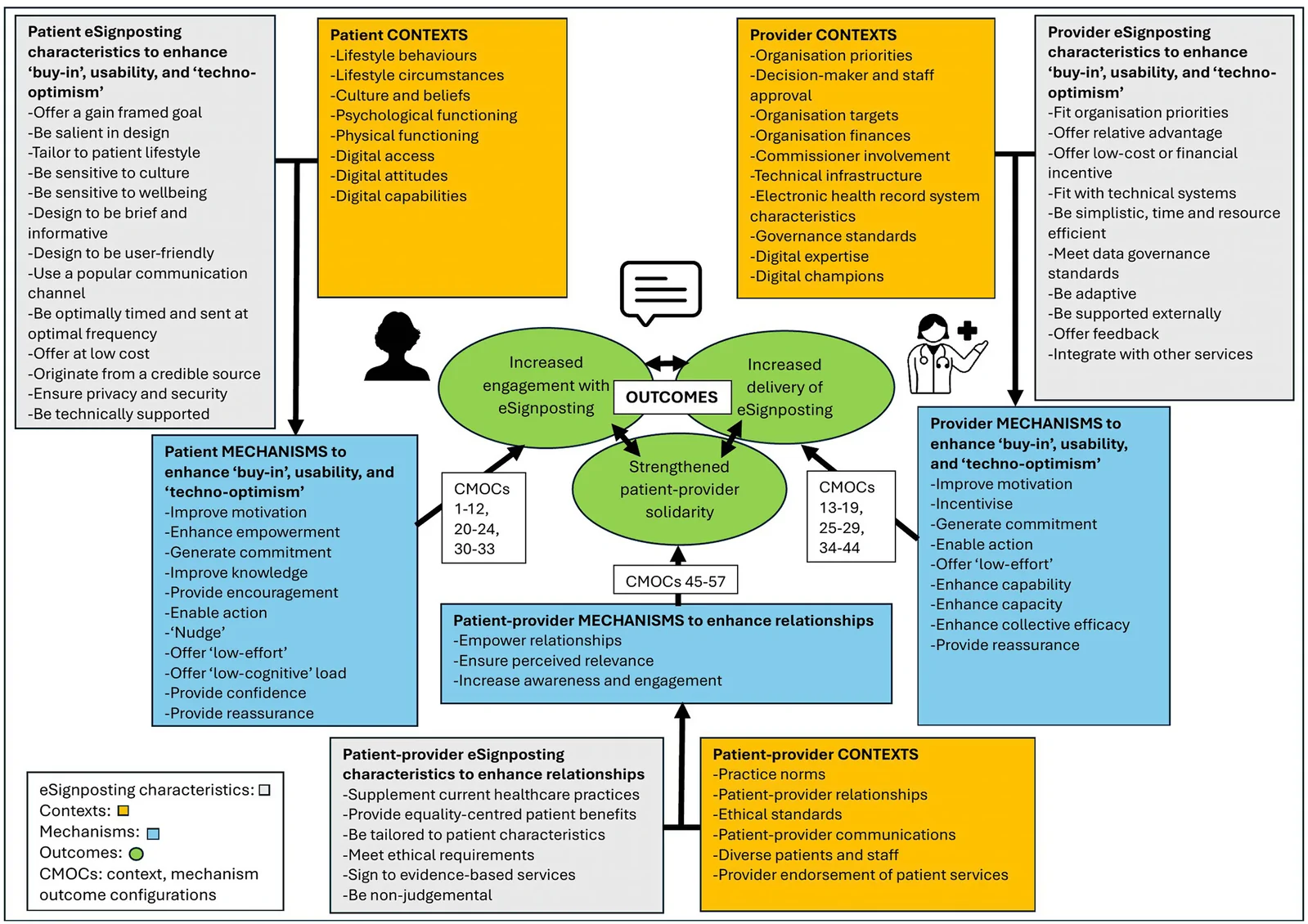
eSign programme theory.

#### Patient ‘buy-in’ to eSignposting.

Patient ‘buy-in’ to eSignposting was an important theme. Key contextual factors were lifestyle behaviours, cultural practices, beliefs, comprehension, emotional wellbeing, financial circumstances, and lifestyle circumstances. Overall, eSignposting messages deemed to generate greater motivation for engagement were those where content was gain-framed (positively worded) and where a simple achievable goal was stipulated which fitted with patient behaviours [12–14,34,35,42,44,48,51,54,58,65]. Enhancing message salience, by using striking wording or images to draw the eye, was also likely to increase motivation to engage. A good fit with an individual’s social norms, e.g., altruistic actions, may additionally aid patient motivation [52,58,76]. Messages predominantly used the English language which was believed limiting for non-English speakers, with ethnic minorities thought to be alienated in some cases, in part due to a history of mistrust with medical systems [36,46,49,59,73,75]. Where message content was sensitive to native language and cultural beliefs, patients were empowered to engage with eSignposting [47,53,54,62]. Messages which were autonomy supporting were identified as increasing commitment to engage [34,39,41,44–46,48,51,52,57,65,67,69]. Other positive attributes for message content were using clear and simple instructions to address health literacy barriers and to increase patient comprehension to maximise engagement [12,35–37,39,40,42,45,51,53,54,56,58,65,69,75]. PPI representatives raised the importance of patient emotional state and mental wellbeing as contextual factors. This was supported by the literature where mechanisms relating to anxiety and feelings of failure were evident in reducing engagement [12,34,35,45,49,75]. Patients would be more likely to engage where messages are sensitive to wellbeing, e.g., minimising anxiety [12,34,35,42,45,49,75]. Two studies [12,76] referred to patient physical functioning, highlighting limitations in eyesight or manual dexterity, suggesting more patients would be enabled to engage if messages are made accessible.

In the context of patient financial circumstances, particularly for individuals of low income, keeping messages and access to services at low cost and making costs/ incentives explicit was more likely to enable participation [12,34,49,51,54,62,77]. Where messages and services offered flexibility and convenience, this enabled patients with busy lifestyles, or with barriers to service access, to engage [12,33,41–43,49–52,55,58,65,74]. If messages were sent at times which complemented patient lifestyle or offered flexibility in time of response, then patients would be encouraged to engage with messages [12,41,43,47,50,51,55,64,75]. Timing to coincide with health promotion campaigns or at times of resolutions, e.g., New Year, may encourage engagement [12,42,77]. Frequency of messages was also identified as important to complement patient lifestyle. Optimal frequency is likely to vary based on the intensity of the intervention, with more ‘nudging’ required for behaviours which require more involved behaviour change, e.g., appointments, and less frequent messages where alert fatigue and feelings of ‘bombardment’ may occur [12,13,33,34,41,43,44,46,47,50,51,53,56].

#### Provider ‘buy-in’ to eSignposting.

Important contextual factors for provider ‘buy-in’ were organisational priorities, staff perceptions of eSignposting, finances, incentives and support from commissioners. Where organisational priorities included a focus on cancer prevention interventions, staff were more committed to delivering eSignposting messages [12,13,33,50,51,53,57,59,71]. We also identified commitment to be a mechanism for ‘buy-in’ from decision makers. If key decision-makers were involved in the implementation of eSignposting, they were more likely to accept and approve strategies thus increasing implementation success [12,39,41,75,77]. Just one study, Khadjesari et al [12], referred to perceptions of wider staff, finding positive perceptions of relative advantage. We hypothesised that perceiving relative advantage was more likely to motivate staff to deliver eSignposting messages, although this CMO configuration was limited to only one study. Where organisational finances were able to accommodate eSignposting, because this offered low comparative cost, staff were more enabled to deliver messages [12–14,33,34,37–39,41,42,46–49,51–56,59,70]. Incentives and targets for patient reach using digital healthcare may further strengthen finances and incentivise staff [37,39,57,71,72]. However, other studies referred to reimbursement being higher for in-person visits and by offering remote support in some cases, eSignposting may reduce use of in-person services, thus reducing finance available to services [12,34,42]. Linked to reimbursement, El-Osta et al [41] emphasised the importance of collaborative work with commissioners to ensure a co-ordinated approach to enable resourcing for eSignposting. Houston et al [44] was the only study which referred to staff awareness of patient benefit, finding increased awareness to be a facilitating factor. We surmised that improved awareness of the benefits that patients are being signposted to would increase staff motivation to deliver eSignposting, although evidence for this CMO configuration was limited. Panel members concurred with the importance of provider ‘buy-in’, identifying particularly with the impact of incentives and the financial environment.

#### Patient perceived system usability.

Usability related to having suitable digital access and capacity to use digital services. Studies linked success to using popular modes of communication, with SMS text and telephone often reported as most popular [12,39,46,47,50,54,57,65]. Favourable outcomes were also linked to making use of more than one communication channel, or more than one type of service intervention [14,34,36,37,39,41,42,45,48,51,52,54–57,59,68]. This related to concerns around particular patient groups being less likely to access digital services or engage with portals, such as older patients, those with lower income, and minorities. Promising characteristics for message content were use of messages that were user-friendly [12,35,39,40,42,45,59], easy to action, e.g., ‘one-click’ [12,34,51,52,64], with brief and simplistic content to address limited health literacy [12,34,35,40,45,46,57,64,73,76]. Success was lower where extra steps were required, such as patient log-in [33,46,56]. Stakeholders also emphasised the importance of making the journey quick and easy for patients. Mechanisms to increase engagement with eSignposting messages included enablement, ‘low effort’ and ‘low cognitive load’. If patient capabilities can be enhanced by using a popular communication mode and a user-friendly approach, these mechanisms are likely to be activated and patients will be more likely to engage with eSignposting messages.

#### Provider perceived system usability.

For providers, usability related to integration with current systems, adaptability for different systems and future changes, accuracy of systems, ease of use, and time and resource efficiency. Where systems were robust, staff were enabled to deliver eSignposting messages, but implementation barriers could occur where systems were less robust [12,14,42,50,59]. Most studies highlighted limitations in system accuracy to be a barrier. Health status information and contact details were often incomplete or out of date, and details such as language preference could be missing [12,14,37,39,40,43,46–50,53–57,64]. There is an opportunity to use algorithms to circumvent this, or for eSignposting to reduce missing data by obtaining details concurrently with signposting [37,46]. Ease of system use was reported as a facilitator where present, or a barrier where there were complexities in systems [12,55,68,70,72]. Success was more likely where eSignposting was time and resource efficient [34,37,39,42,44,48,51,53,55,75,77], but two studies cautioned that full automation would require specialists and could be costly [46,47]. In line with patient mechanisms for system usability, we identified enablement and ‘low effort’ to be common mechanisms for providers. The theme of system usability and compatibility with varied dynamic systems resonated particularly well with panel members. We found that if eSignposting is quick, easy to use, and integrates into varied technical systems, then providers will be more likely to send eSignposting messages.

#### Patient ‘techno-optimism’.

We used the term ‘techno-optimism’ [79] to reflect positive beliefs and attitudes towards technology [79]. Attitudes were optimistic [12,33,69,75] or pessimistic [33,49,58,64], and linked to familiarity with technology. COVID-19 was noted to have increased preference for and familiarity with digital services [33,75]. Negativities appeared to arise in the context of scepticism to digitisation including a preference for in-person services, or concerns over privacy, security and trustworthiness of messages. eSignposting characteristics needed to increase positivity were identified to be a favourable and simple design to increase ‘onboarding’ [35] with digital health, with the provision of digital support if necessary. We identified optimism and confidence as mechanisms for increasing engagement with eSignposting messages. It emerged that clearly identifying healthcare providers as senders was important to foster trust and to reassure patients that the messages were authentic, private and secure [12,44,75,76]. In studies where the sender was not identified or where there was a lack of awareness of message sending, the mechanism of reassurance was not triggered, resulting in patient concerns [64,74]. Reassurance of message authenticity and clarification of message sender were also highlighted as key by our PPI representatives.

#### Provider ‘techno-optimism’.

‘Techno-optimism’ of providers related to systems meeting governance standards (e.g., data control) [12,13,39,41,49,70,77], flexibility to incorporate new technology and integration with existing workflow systems [34,39,41,51,52,54,57,59,71–73], sufficient resourcing [42,50,53,54,69,72], training for system use [34,44,57], previous digital experience [33,34,39,40,44,51,60,72], and feedback of eSignposting impact [12,34,44,50,75,77]. Some studies noted the importance of building in checkpoints and aligning systems with existing data governance structures to support integration [39,41,49,72,77]. Good partnerships with external organisations and centralisation of systems were also important to support integration within and between organisations [12,14,36,39,41,44,50,54]. A culture of embracing new technologies and promotion by digital champions were identified as positive factors to promote eSignposting [12,39,44,59,75]. Challenges arose from increased workload and staff turnover [12,71], and there were some reports of lack of system knowledge and uncertainties regarding completion of training [12,41,43]. Aligning with patient ‘techno-optimism’, we identified reassurance, confidence, optimism and enthusiasm as mechanisms for success. We also identified capacity, capability, collective efficacy and adaptability to be important in increased likelihood of providers delivering messages. The importance of ease and flexibility to incorporate new technical systems, together with the ability to champion these, resonated well with our panel members. An eSignposting system that is time and resource efficient appears key to enhancing ‘techno-optimism’ and implementation success.

#### Patient-professional solidarity.

If eSignposting complements current healthcare norms by acting as a supplement to usual practice then patient-provider relationships would be empowered and eSignposting would have greater acceptability [12–14,34,37,39,41,42,47,50,51,55,57,59,69,71,73]. However, our panel members and the literature also raised concerns of inadvertently increasing patient inequalities through the use of digital messages, due to digital divide. Older patients, low-income groups, and minorities may be disadvantaged, e.g., portals were less likely to be accessed by minority groups [36,48], and messages were predominantly written in English. A design to include marginalised groups and to promote equality-centred patient benefit would further empower patient-provider relationships [12,35,36,39,41,42,50,51,54,56,57,65,75]. Tailoring messages to patient needs and preferences may also trigger the mechanism of empowerment, if tailoring could be designed in a way that would not add burden to providers [12,13,34,35,39–41,43–46,49–51,54,58,60]. Personalising messages to include patient names might strengthen patient-provider relationships through the mechanism of perceived relevance [34,44,57,58,65,76], although one study raised privacy concerns as a potential inadvertent outcome of this [12]. If patient ethics, in particular adding an opt-out of data use option are met, then patients and providers will be less resistant and more accepting of eSignposting [12,14,36,49,50,54,58,60]. Patient opt-out was preferred over patient opt-in, to reduce an extra step of enrolment. Two studies made reference to suitability of eligibility criteria such as BMI ‘cut-off’, noting eligibility is not constant but changes over time [66], and that messages may not be suitable for those with borderline eligibility [34]. Where eSignposting messages can be well matched to eligibility criteria, we hypothesised that patients would perceive greater relevance of messages. In studies which raised awareness of eSignposting messages, providers and patients were better prepared for messages [13,34,52,53,70,72,76,78]. Whereas for studies where awareness was not raised, staff reported an interruption to patient interactions [64] and patients raised doubts of message authenticity [74]. There was greater acceptability of eSignposting where diverse groups of patients and/or providers had contributed to the design of messages and processes [12,14,34,39–41,44,46,48,50,51,54,65,68,77]. Acceptability was also enhanced by the use of evidence-based interventions [12,33–35,44,47,50–52,73]; and by endorsement from providers [12,14,34,37,41,45,48,50,51,53,54,56,64] perceived as providing reassurance. Regarding timing of message receipt in relation to patient appointments, we identified two countertheories: If messages are linked to a patient appointment, then patients and providers will be motivated, and eSignposting will have greater acceptability [12,13,39,40,44,47,51,62,69,74]. On the other hand, if messages are not linked to patient appointments, then patients and providers will experience less burden, and eSignposting will have greater acceptability [12,39,41,42,47,50,57,69,77]. Whether messages were sent as part of patient appointments or not, presenting messages non-judgementally served to both reduce stigma and increase eSignposting acceptability [12,34,37,49,50,77].

## Discussion

We included 30 studies in our realist review and used this literature in conjunction with stakeholder panel and patient and public involvement to develop a novel programme theory for electronic signposting to cancer prevention interventions. We identified 57 CMO configurations across seven theoretical areas: patient, and provider ‘buy-in’; patient, and provider perceived system usability; patient, and provider ‘techno-optimism’; and patient-professional relationships. We found that eSignposting to a variety of different cancer interventions, using various modes, e.g., portal, email, or SMS texts can generate good levels of engagement for diverse patient groups. We detected no clear patterns relating to timing and frequency of messages. For patients, implementation success related to memorable messages tailored to match patient behavioural, medical, lifestyle and cultural characteristics. Using positive framing and setting clear goals was important. Use of popular communication channel(s) and ensuring messages were clear, simple and easy to action was central to improved usability. For example, a message with a ‘one-click’ function would likely increase engagement in comparison to a message requiring multiple steps. Patient positivity could be enhanced by providing technical support and reassurance that messages were trustworthy and secure. For providers, ‘buy-in’ related to a good fit with organisational priorities and finances, and involvement of senior teams and champions to motivate staff. eSignposting worked best in situations where robust technical systems were in place and where eSignposting was time and resource efficient. Meeting data governance standards was crucial. For example, a technical system which preserves security and privacy of patient data, would likely increase engagement, in comparison to a system where data governance standards are not met. ‘Techno-optimism’ was present where there was previous technical experience, or a progressive attitude towards technology. Maintenance of good patient-professional relationships was also key, achieved through a focus on equality-centred patient benefit.

We identified two interesting counter theories regarding message timing. On the one hand, sending messages at a time linked to a healthcare appointment might increase motivation to engage with messages through momentum of dialogue; whilst on the other hand, sending messages at a time outside of a healthcare appointment might increase engagement by reduced burden to patients and providers through reduced time pressure and added convenience. The former may work best in contexts where patients are more likely to have health professional contact, e.g., patients with chronic conditions. Whilst the latter may work best in contexts where health professionals are under severe time pressure, or in areas where patients are less likely to seek care, e.g., geographically remote locations.

Investments in information systems are recommended to improve the economic feasibility and accessibility of cancer prevention services [2]. Digital messaging is increasingly used in healthcare settings, a trend boosted in part by the COVID-19 pandemic [80]. Electronic signposting and digital interventions have the potential to promote health in a manner that could reduce costs and pressures on healthcare services and increase convenience for patients [81], a high priority in the context of an expanding and aging population. However, implementation of eSignposting must be optimised for this strategy to work effectively and to reduce disparities, rather than adding to stigma and creating digital divide [82]. Our programme theory provides insight into maximising implementation for eSignposting success. Whilst study heterogeneity limited ability to identify quantitative determinant affects, common facilitators for success were driving increased motivation to change through meeting priorities and perceived advantage, and the use of low-cost, low burden solutions with assured data governance. These drivers align with the technology, value proposition and adopter domains of the NASSS Framework [16], and appeared to play a greater role than specifics of cancer type or cancer prevention intervention. eSignposting which implements these drivers is more likely to be successful. Other digital health realist reviews concur with our findings, reporting alignment with priorities, management engagement, training, workload integration, and audit and feedback to be effective implementation strategies for organisations [17,21,22]. Our findings also reflect the wider literature of signposting which uses face-to-face or other referral methods. A realist review by Cantrell et al 2024 found that training and support, and tailoring of services to patients were important factors [83] Digital health reviews have highlighted the importance of factors including ease of use, flexibility and personalisation, addressing confidentiality concerns, and maintenance of existing patient-provider relationships [17,84]. Maintaining patient-provider relationships might be of greatest importance for marginalised groups [85]. Key causal mechanisms for digital interventions identified in the literature include motivation and perceived usefulness [17], which our review also identified as themes. We found that eSignposting worked through a variety of different mechanisms to generate outcomes, evidence that eSignposting is a complex intervention which must be carefully implemented and designed with diverse patient and provider input.

Strengths of our review lie in the thoroughness of our approach. We systematically searched multiple databases, grey literature, and involved stakeholders, including PPI. We also wrote to authors of included studies for additional information. Our independent screening for study inclusion and data extraction exceeded the 10% rate considered typical for realist reviews [86] which we believe adds to the robustness of our findings. We used a combination of three theoretical frameworks: NASSS [16]; MINDSPACE [31]; and COM-B [32] to aid identification of likely pathways and strengthen our refined programme theory.

Limitations to the review relate to the lack of detail of context, mechanisms and outcomes present within some studies and where we were unsuccessful in gaining additional information from authors, despite our best efforts. There were relatively few qualitative studies, or studies which provided detailed process evaluation or cost analysis. Additionally, some trial studies were pragmatic in nature, with few studies including an appropriate control group, and some studies were limited by small sample sizes. Further, the wide variation in how reach and engagement were defined within individual included studies, limited comparisons of outcomes between studies. Using patient portals to send messages, resulted in a bias towards inclusion of patients who were already registered on portals and were therefore more inclined to accept technology and to be engaged with services. Some studies required ethical approval, whilst others were conducted as quality improvement initiatives which resulted in some differences in types of patients responding. Furthermore, most studies were conducted in the United States where healthcare technology is more advanced in comparison to other countries. As such, our programme theory may lack transferability to healthcare systems in other countries, particularly where digital technology and skills are less well developed. In other countries and healthcare contexts, it is likely that outcomes would be less successful where digital systems are less robust and where staff do not receive sufficient training. With regard to our eligibility criteria, we focused on adults 18 years or over, since we believed the mechanisms identified may operate differently in children. However, this choice may limit the use of the theory for longer-term cancer prevention strategies, including cancer prevention in children. Finally, we recognise that our programme theory could be further tested and enhanced, and recognise that other models might conceptualise mechanisms differently [87].

Future empirical eSignposting studies would benefit from more detailed process evaluation, qualitative insight exploring in-depth patient-provider views, assessment of cost-effectiveness, equity impact and long-term outcomes. There is also a critical need for empirical studies in non-USA countries, and countries where technology is less well developed, such as low-and-middle-income countries, to develop the programme theory, identify any changes to CMOs, and to establish how the theory could be operationalised in countries outside of the USA. We recommend additional research to establish more precise recommendations within particular contexts, for example, the minimal technical requirements for eSignposting to succeed. We identified two counter theories relating to advantages of sending messages at a time linked to a healthcare appointment (increased motivation) versus advantages of sending messages at a time not linked to a healthcare appointment (lower burden). Primary research to test these two strategies could extend and refine the programme theory. Future research to bridge the fields of realist research and implementation science, would aid the communication of programme theories to stakeholders, and enable adoption of more successful strategies within particular contexts.

## Conclusion

We have developed a novel programme theory for eSignposting to cancer prevention interventions which extends current literature and can guide and support stakeholders to implement eSignposting programmes. We found that eSignposting using a variety of modes and sent at various frequencies could generate good engagement for a variety of patient groups. Use of salient messages with wording closely matched with patient characteristics increased ‘buy-in’. Use of simple, ‘low-effort’ messages using popular communication channels improved patient usability. Supporting technical abilities and reassurance that messages were authentic and secure increased patient optimism and uptake of eSignposting. For health professional providers, a good fit with organisational priorities, finances, and ‘buy-in’ from senior teams was important. eSignposting worked best in a context of robust technical systems and where eSignposting was resource efficient. Previous technical experience or a progressive attitude towards technology increased optimism. Ensuring a context of continued patient-professional relationships was also important, particularly regarding patient equity. eSignposting must be optimised to reduce disparities, rather than creating digital divide. We recommend policymakers and healthcare providers implement electronic signposting in ways that would increase equality-centred patient benefit, to reduce cancer and cancer disparities.

## Supporting information

S1 Table RAMESES checklist of items required when reporting a realist synthesis. (DOCX)

S2 Table eSign search strategies. (DOCX)

S3 Table eSign data extraction. (XLSX)

S4 Table Refined programme theory of electronic signposting to cancer prevention interventions. (DOCX)
