# Supplementary material for: Implementation of electronic signposting to interventions that prevent cancer: A realist review
Source: PLOS Digit Health. 2026 Oct 5;5(10):e0001770. doi: 10.1371/journal.pdig.0001770 (PMC13637878; doi:10.1371/journal.pdig.0001770)
Supplement: Supplementary file 2 [file pdig.0001770.s002.docx]

**S2 Tables: eSign search strategies**

AMED eSign final search ran 27.09.24

| **#** | **Query** | **Results** |
| --- | --- | --- |
| S1 | (((((ZU "electronic health records")) or ((ZU "electronic medical records"))) or ((ZU "medical records"))) or ((ZU "patient portals"))) or ((ZU "medical records problem oriented")) | 457 |
| S2 | (((((ZU "text messaging")) or ((ZU "electronic mail"))) or ((ZU "telephone"))) or ((ZU "telemedicine"))) or ((ZU "referral and consultation")) | 2,576 |
| S3 | ((ZU "human papillomavirus")) or ((ZU "hepatitis b")) | 16 |
| S4 | (((ZU "vaccines")) or ((ZU "vaccination"))) or ((ZU "immunization")) | 355 |
| S5 | S3 AND S4 | 2 |
| S6 | ((((((((((((((((((((ZU "early diagnosis")) or ((ZU "health behavior"))) or ((ZU "health education"))) or ((ZU "health promotion"))) or ((ZU "smoking cessation"))) or ((ZU "smoking"))) or ((ZU "tobacco use cessation devices"))) or ((ZU "overweight"))) or ((ZU "obesity"))) or ((ZU "weight loss"))) or ((ZU "healthy diet"))) or ((ZU "healthy lifestyle"))) or ((ZU "exercise"))) or ((ZU "alcohol drinking"))) or ((ZU "sunburn"))) or ((ZU "sunscreening agents"))) or ((ZU "sunbathing"))) or ((ZU "vaginal smears"))) or ((ZU "mammography"))) or ((ZU "cancer vaccines")) | 16,424 |
| S7 | S5 OR S6 | 16,426 |
| S8 | S1 AND S7 | 9 |
| S9 | S1 AND S2 | 15 |
| S10 | S2 AND S7 | 118 |
| S11 | S8 OR S9 OR S10 | 142 |
| S12 | TX ("electronic health* record*" OR "patient* portal*" OR "web portal*" OR "e-portal*" OR eportal* OR "electronic portal*" OR "medical record* system*" OR "personal health record*" OR (patient* N1 portal*) OR "electronic medical record*" OR "electronic patient* record*" OR "computer* patient* record*" OR "electronic patient* medical record*" OR "digital health record*" OR "digital patient* record*" OR "computer* medical record*" OR "digital medical record*" OR "digital patient* medical record*" OR "computer* health record*" OR "computer* patient* medical record*") | 334 |
| S13 | signpost* OR "e-refer*" OR erefer* OR "text messag*" OR "short message service*" OR "multimedia messaging service*" OR "rich communication service*" OR "instant messag*" OR "push notification*" OR "app notification*" OR "app messag*" OR email* OR "e-mail*" OR "electronic mail*" OR "electronic-mail*" OR eHealth OR "e-Health" OR mHealth OR "m-Health" OR "mobile-health" OR "one-click" OR "opt-out" | 713 |
| S14 | (digital* OR electronic* OR mobile* OR "mobile phone*" OR "cell phone*" OR telephone* OR phone* OR smartphone* OR "smart phone*" OR smartwatch* OR "smart watch*" OR automat* OR virtual OR video* OR online OR internet OR computer* OR remote OR "provider-initiated" OR proactive*) N1 (messag* OR refer* OR screen* OR reminder* OR promot* OR communicat* OR text* OR intervention* OR appointment* OR apps OR applications) | 1,142 |
| S15 | SMS N3 (text* OR messag* OR reminder* OR intervention* OR educat* OR appointment* OR refer* OR promot* OR communicat*) | 26 |
| S16 | MMS N3 (text* OR messag* OR reminder* OR intervention* OR educat* OR appointment* OR refer* OR promot* OR communicat*) | 3 |
| S17 | RCS N3 (text* OR messag* OR reminder* OR intervention* OR educat* OR appointment* OR refer* OR promot* OR communicat*) | 1 |
| S18 | health N1 (behavior* OR behaviour* OR promot* OR educat* OR intervention*) | 5,473 |
| S19 | (behavior OR behaviour) N1 (change* OR changing OR promot* OR educat* OR intervention*) | 739 |
| S20 | cancer* N1 (risk* OR prevent* OR screen*) | 491 |
| S21 | (smoking OR smoker* OR tobacco OR cigarette* OR "nicotine replacement*") N1 (cessation OR cease OR ceasing OR quit* OR prevent* OR relapse OR behav* OR educat* OR treatment* OR therapy OR therapies OR intervention*) | 467 |
| S22 | (obese OR obesity OR BMI OR weight OR overweight) N1 (prevent* OR educat* OR risk* OR reduce* OR reducing OR reduction OR minimiz* OR minimis* OR decreas* OR lower* OR less* OR change* OR changing OR improve* OR improving OR better OR gain OR loss* OR lose OR losing OR excess* OR intervention*) | 2,759 |
| S23 | (diet* OR eat*) N1 (health* OR behav* OR change* OR changing OR improve* OR improving OR better OR educat* OR promot* OR intervention*) | 712 |
| S24 | ("physical activity" OR exercise) N1 (health* OR behav* OR change* OR changing OR improve* OR improving OR better OR educat* OR promot* OR intervention*) | 3,688 |
| S25 | (drink* OR alcohol*) N1 (drinking OR heavy OR risk* OR excess* OR consum* OR cessation OR cease OR ceasing OR quit* OR reduce* OR reducing OR reduction OR limit* OR free OR minimiz* OR minimis* OR decreas* OR lower* OR less OR lessen* OR "cut* back" OR "cut* down" OR change* OR changing OR improve* OR improving OR better OR behav* OR educat* OR prevent* OR intervention*) | 637 |
| S26 | (sunscreen OR suncream) N1 ("use of" OR using OR apply* OR application OR intervention* OR promot* OR educat*) | 2 |
| S27 | "sun protection" OR "sun exposure" OR "suncream use" OR "sunscreen use" OR "sun safety" OR "sun smart" OR sunsmart OR sunburn OR "sun damage" OR "UV radiation" OR "UV exposure" OR "UV protection" | 77 |
| S28 | "HPV vaccin*" OR "papillomavirus vaccin*" OR "Hep* B vaccin*" OR "HBV vaccin*" OR "HPV screening*" OR "papillomavirus screening*" | 15 |
| S29 | "cervical screening*" OR "vaginal smear*" OR "cervical smear*" OR mammogra* OR "breast screening*" OR "bowel screening*" OR "colorectal screening*" OR "CRC screening*" | 78 |
| S30 | S13 OR S14 OR S15 OR S16 OR S17 | 1,736 |
| S31 | S18 OR S19 OR S20 OR S21 OR S22 OR S23 OR S24 OR S25 OR S26 OR S27 OR S28 OR S29 | 13,424 |
| S32 | S12 AND S30 AND S31 | 2 |
| S33 | S11 OR S32 | 143 |

Cinahl eSign final search ran 27.09.24

|  | **Query** | **Results** |
| --- | --- | --- |
| S1 | (MH "Patient Portals") OR (MH "Electronic Health Records+") OR (MH "Patient Record Systems") OR (MH "Clinical Information Systems") OR (MH "Medical Records, Personal") OR (MH "Medical Record Linkage") | 48,389 |
| S2 | (MH "Text Messaging") OR (MH "Telehealth") OR (MH "Telemedicine") OR (MH "Telephone+") OR (MH "Email") OR (MH "Instant Messaging") OR (MH "Interactive Voice Response Systems") OR (MH "Digital Health") OR (MH "Referral and Consulation") | 68,959 |
| S3 | (MH "Early Detection of Cancer") OR (MH "Health Behavior") OR (MH "Health Education") OR (MH "Health Promotion") OR (MH "Smoking Cessation") OR (MH "Smoking Cessation Programs") OR (MH "Smoking") OR (MH "Obesity") OR (MH "Weight Reduction Programs") OR (MH "Diet, Reducing") OR (MH "Life Style Changes") OR (MH "Life Style, Sedentary") OR (MH "Exercise") OR (MH "Alcohol Drinking") OR (MH "Sun Protective Behavior") OR (MH "Sunscreening Agents") OR (MH "Sunburn") OR (MH "Papillomavirus Vaccine") OR (MH "Cancer Vaccines") OR (MH "Hepatitis B Vaccines") OR (MH "Vaccination Promotion") OR (MH "Cervical Smears") OR (MH "Mammography") | 446,288 |
| S4 | S1 AND S2 AND S3 | 98 |
| S5 | TX ("electronic health* record*" OR "patient* portal*" OR "web portal*" OR "e-portal*" OR eportal* OR "electronic portal*" OR "medical record* system*" OR "personal health record*" OR (patient* N1 portal*) OR "electronic medical record*" OR "electronic patient* record*" OR "computer* patient* record*" OR "electronic patient* medical record*" OR "digital health record*" OR "digital patient* record*" OR "computer* medical record*" OR "digital medical record*" OR "digital patient* medical record*" OR "computer* health record*" OR "computer* patient* medical record*") | 114,355 |
| S6 | signpost* OR "e-refer*" OR erefer* OR "text messag*" OR "short message service*" OR "multimedia messaging service*" OR "rich communication service*" OR "instant messag*" OR "push notification*" OR "app notification*" OR "app messag*" OR email* OR "e-mail*" OR "electronic mail*" OR "electronic-mail*" OR eHealth OR "e-Health" OR mHealth OR "m-Health" OR "mobile-health" OR "one-click" OR "opt-out" | 56,887 |
| S7 | (digital* OR electronic* OR mobile* OR "mobile phone*" OR "cell phone*" OR telephone* OR phone* OR smartphone* OR "smart phone*" OR smartwatch* OR "smart watch*" OR automat* OR virtual OR video* OR online OR internet OR computer* OR remote OR "provider-initiated" OR proactive*) N1 (messag* OR refer* OR screen* OR reminder* OR promot* OR communicat* OR text* OR intervention* OR appointment* OR apps OR applications) | 51,425 |
| S8 | SMS N3 (text* OR messag* OR reminder* OR intervention* OR educat* OR appointment* OR refer* OR promot* OR communicat*) | 1,062 |
| S9 | MMS N3 (text* OR messag* OR reminder* OR intervention* OR educat* OR appointment* OR refer* OR promot* OR communicat*) | 37 |
| S10 | RCS N3 (text* OR messag* OR reminder* OR intervention* OR educat* OR appointment* OR refer* OR promot* OR communicat*) | 42 |
| S11 | health N1 (behavior* OR behaviour* OR promot* OR educat* OR intervention*) | 267,211 |
| S12 | (behavior OR behaviour) N1 (change* OR changing OR promot* OR educat* OR intervention*) | 26,938 |
| S13 | cancer* N1 (risk* OR prevent* OR screen*) | 64,514 |
| S14 | (smoking OR smoker* OR tobacco OR cigarette* OR "nicotine replacement*") N1 (cessation OR cease OR ceasing OR quit* OR prevent* OR relapse OR behav* OR educat* OR treatment* OR therapy OR therapies OR intervention*) | 46,311 |
| S15 | (obese OR obesity OR BMI OR weight OR overweight) N1 (prevent* OR educat* OR risk* OR reduce* OR reducing OR reduction OR minimiz* OR minimis* OR decreas* OR lower* OR less* OR change* OR changing OR improve* OR improving OR better OR gain OR loss* OR lose OR losing OR excess* OR intervention*) | 116,859 |
| S16 | (diet* OR eat*) N1 (health* OR behav* OR change* OR changing OR improve* OR improving OR better OR educat* OR promot* OR intervention*) | 58,912 |
| S17 | ("physical activity" OR exercise) N1 (health* OR behav* OR change* OR changing OR improve* OR improving OR better OR educat* OR promot* OR intervention*) | 37,549 |
| S18 | (drink* OR alcohol*) N1 (drinking OR heavy OR risk* OR excess* OR consum* OR cessation OR cease OR ceasing OR quit* OR reduce* OR reducing OR reduction OR limit* OR free OR minimiz* OR minimis* OR decreas* OR lower* OR less OR lessen* OR "cut* back" OR "cut* down" OR change* OR changing OR improve* OR improving OR better OR behav* OR educat* OR prevent* OR intervention*) | 65,941 |
| S19 | (sunscreen OR suncream) N1 ("use of" OR using OR apply* OR application OR intervention* OR promot* OR educat*) | 666 |
| S20 | "sun protection" OR "sun exposure" OR "suncream use" OR "sunscreen use" OR "sun safety" OR "sun smart" OR sunsmart OR sunburn OR "sun damage" OR "UV radiation" OR "UV exposure" OR "UV protection" | 6,242 |
| S21 | "HPV vaccin*" OR "papillomavirus vaccin*" OR "Hep* B vaccin*" OR "HBV vaccin*" OR "HPV screening*" OR "papillomavirus screening*" | 11,305 |
| S22 | "cervical screening*" OR "vaginal smear*" OR "cervical smear*" OR mammogra* OR "breast screening*" OR "bowel screening*" OR "colorectal screening*" OR "CRC screening*" | 27,717 |
| S23 | S6 OR S7 OR S8 OR S9 OR S10 | 99,722 |
| S24 | S11 OR S12 OR S13 OR S14 OR S15 OR S16 OR S17 OR S18 OR S19 OR S20 OR S21 OR S22 | 614,646 |
| S25 | S5 AND S23 AND S24 | 937 |
| S26 | S4 OR S25 | 993 |

EMBASE eSign final search ran 27.09.24

|  | Searches | Results |
| --- | --- | --- |
| 1 | exp electronic health record/ or exp medical record/ or exp electronic medical record/ or exp electronic patient record/ or problem oriented medical record/ | 357389 |
| 2 | text messaging/ or e-mail/ or *telephone/ or telehealth/ or digital health/ or interactive voice response system/ | 70293 |
| 3 | early cancer diagnosis/ or health behavior/ or health education/ or health promotion/ or smoking cessation/ or smoking reduction/ or smoking prevention/ or "tobacco use"/ or smoking/ or cigarette smoking/ or obesity/ or obesity management/ or weight loss program/ or healthy diet/ or healthy lifestyle/ or exercise/ or drinking behavior/ or sunscreen/ or sunburn/ or sun exposure/ or human papilloma virus vaccine/ or hepatitis B vaccine/ or vagina smear/ or mammography/ or cancer vaccine/ | 1807748 |
| 4 | 1 and 2 and 3 | 387 |
| 5 | ("electronic health* record*" or "patient* portal*" or "web portal*" or "e-portal*" or eportal* or "electronic portal*" or "medical record* system*" or "personal health record*" or (patient* adj2 portal*) or "electronic medical record*" or "electronic patient* record*" or "computer* patient* record*" or "electronic patient* medical record*" or "digital health record*" or "digital patient* record*" or "computer* medical record*" or "digital medical record*" or "digital patient* medical record*" or "computer* health record*" or "computer* patient* medical record*").mp. | 187231 |
| 6 | (signpost* or "e-refer*" or erefer* or "text messag*" or "short message service*" or "multimedia messaging service*" or "rich communication service*" or "instant messag*" or "push notification*" or "app notification*" or "app messag*" or email* or "e-mail*" or "electronic mail*" or "electronic-mail*" or eHealth or "e-Health" or mHealth or "m-Health" or "mobile-health" or "one-click" or "opt-out").ti,ab. | 86813 |
| 7 | ((digital* or electronic* or mobile* or "mobile phone*" or "cell phone*" or telephone* or phone* or smartphone* or "smart phone*" or smartwatch* or "smart watch*" or automat* or virtual or video* or online or internet or computer* or remote or "provider-initiated" or proactive*) adj2 (messag* or refer* or screen* or reminder* or promot* or communicat* or text* or intervention* or appointment* or apps or applications)).ti,ab. | 103119 |
| 8 | (SMS adj4 (text* or messag* or reminder* or intervention* or educat* or appointment* or refer* or promot* or communicat*)).ti,ab. | 3146 |
| 9 | (MMS adj4 (text* or messag* or reminder* or intervention* or educat* or appointment* or refer* or promot* or communicat*)).ti,ab. | 261 |
| 10 | (RCS adj4 (text* or messag* or reminder* or intervention* or educat* or appointment* or refer* or promot* or communicat*)).ti,ab. | 184 |
| 11 | (health adj2 (behavior* or behaviour* or promot* or educat* or intervention*)).ti,ab. | 252896 |
| 12 | ((behavior or behaviour) adj2 (change* or changing or promot* or educat* or intervention*)).ti,ab. | 54746 |
| 13 | (cancer* adj2 (risk* or prevent* or screen*)).ti,ab. | 232107 |
| 14 | ((smoking or smoker* or tobacco or cigarette* or "nicotine replacement*") adj2 (cessation or cease or ceasing or quit* or prevent* or relapse or behav* or educat* or treatment* or therapy or therapies or intervention*)).ti,ab. | 84564 |
| 15 | ((obese or obesity or BMI or weight or overweight) adj2 (prevent* or educat* or risk* or reduce* or reducing or reduction or minimiz* or minimis* or decreas* or lower* or less* or change* or changing or improve* or improving or better or gain or loss* or lose or losing or excess* or intervention*)).ti,ab. | 481438 |
| 16 | ((diet* or eat*) adj2 (health* or behav* or change* or changing or improve* or improving or better or educat* or promot* or intervention*)).ti,ab. | 128752 |
| 17 | (("physical activity" or exercise) adj2 (health* or behav* or change* or changing or improve* or improving or better or educat* or promot* or intervention*)).ti,ab. | 97823 |
| 18 | ((drink* or alcohol*) adj2 (drinking or heavy or risk* or excess* or consum* or cessation or cease or ceasing or quit* or reduce* or reducing or reduction or limit* or free or minimiz* or minimis* or decreas* or lower* or less or lessen* or "cut* back" or "cut* down" or change* or changing or improve* or improving or better or behav* or educat* or prevent* or intervention*)).ti,ab. | 281952 |
| 19 | ((sunscreen or suncream) adj2 ("use of" or using or apply* or application or intervention* or promot* or educat*)).ti,ab. | 2566 |
| 20 | ("sun protection" or "sun exposure" or "suncream use" or "sunscreen use" or "sun safety" or "sun smart" or sunsmart or sunburn or "sun damage" or "UV radiation" or "UV exposure" or "UV protection").ti,ab. | 34585 |
| 21 | ("HPV vaccin*" or "papillomavirus vaccin*" or "Hep* B vaccin*" or "HBV vaccin*" or "HPV screening*" or "papillomavirus screening*").ti,ab. | 30649 |
| 22 | ("cervical screening*" or "vaginal smear*" or "cervical smear*" or mammogra* or "breast screening*" or "bowel screening*" or "colorectal screening*" or "CRC screening*").ti,ab. | 72717 |
| 23 | 6 or 7 or 8 or 9 or 10 | 179564 |
| 24 | 11 or 12 or 13 or 14 or 15 or 16 or 17 or 18 or 19 or 20 or 21 or 22 | 1566790 |
| 25 | 5 and 23 and 24 | 1180 |
| 26 | 4 or 25 | 1467 |

ERIC eSign final search ran 27.09.24

|  | **Searches** | **Results** |
| --- | --- | --- |
| S1 | DE "Case Records" or DE "Databases" or DE "Online Systems" | 16,398 |
| S2 | DE "Physician Patient Relationship" OR DE "Electronic Mail" OR DE "Telecommunications" OR DE "Handheld Devices" | 23,548 |
| S3 | DE "Clinical Diagnosis" OR DE "Diagnostic Tests" OR DE "Screening Tests" OR DE "Cancer" OR DE "Health Behavior" OR DE "Health Education" OR DE "Health Promotion" OR DE "Smoking" OR DE "Obesity" OR DE "Wellness" OR DE "Exercise" OR DE "Alcoholism" OR DE "Drinking" OR DE "Immunization Programs" | 60,292 |
| S4 | S1 AND S2 AND S3 | 12 |
| S5 | TX ("electronic health* record*" OR "patient* portal*" OR "web portal*" OR "e-portal*" OR eportal* OR "electronic portal*" OR "medical record* system*" OR "personal health record*" OR (patient* N1 portal*) OR "electronic medical record*" OR "electronic patient* record*" OR "computer* patient* record*" OR "electronic patient* medical record*" OR "digital health record*" OR "digital patient* record*" OR "computer* medical record*" OR "digital medical record*" OR "digital patient* medical record*" OR "computer* health record*" OR "computer* patient* medical record*") | 456 |
| S6 | signpost* OR "e-refer*" OR erefer* OR "text messag*" OR "short message service*" OR "multimedia messaging service*" OR "rich communication service*" OR "instant messag*" OR "push notification*" OR "app notification*" OR "app messag*" OR email* OR "e-mail*" OR "electronic mail*" OR "electronic-mail*" OR eHealth OR "e-Health" OR mHealth OR "m-Health" OR "mobile-health" OR "one-click" OR "opt-out" | 555,779 |
| S7 | (digital* OR electronic* OR mobile* OR "mobile phone*" OR "cell phone*" OR telephone* OR phone* OR smartphone* OR "smart phone*" OR smartwatch* OR "smart watch*" OR automat* OR virtual OR video* OR online OR internet OR computer* OR remote OR "provider-initiated" OR proactive*) N1 (messag* OR refer* OR screen* OR reminder* OR promot* OR communicat* OR text* OR intervention* OR appointment* OR apps OR applications) | 34,593 |
| S8 | SMS N3 (text* OR messag* OR reminder* OR intervention* OR educat* OR appointment* OR refer* OR promot* OR communicat*) | 158 |
| S9 | MMS N3 (text* OR messag* OR reminder* OR intervention* OR educat* OR appointment* OR refer* OR promot* OR communicat*) | 12 |
| S10 | RCS N3 (text* OR messag* OR reminder* OR intervention* OR educat* OR appointment* OR refer* OR promot* OR communicat*) | 49 |
| S11 | health N1 (behavior* OR behaviour* OR promot* OR educat* OR intervention*) | 49,597 |
| S12 | (behavior OR behaviour) N1 (change* OR changing OR promot* OR educat* OR intervention*) | 20,766 |
| S13 | cancer* N1 (risk* OR prevent* OR screen*) | 432 |
| S14 | (smoking OR smoker* OR tobacco OR cigarette* OR "nicotine replacement*") N1 (cessation OR cease OR ceasing OR quit* OR prevent* OR relapse OR behav* OR educat* OR treatment* OR therapy OR therapies OR intervention*) | 1,468 |
| S15 | (obese OR obesity OR BMI OR weight OR overweight) N1 (prevent* OR educat* OR risk* OR reduce* OR reducing OR reduction OR minimiz* OR minimis* OR decreas* OR lower* OR less* OR change* OR changing OR improve* OR improving OR better OR gain OR loss* OR lose OR losing OR excess* OR intervention*) | 2,264 |
| S16 | (diet* OR eat*) N1 (health* OR behav* OR change* OR changing OR improve* OR improving OR better OR educat* OR promot* OR intervention*) | 2,318 |
| S17 | ("physical activity" OR exercise) N1 (health* OR behav* OR change* OR changing OR improve* OR improving OR better OR educat* OR promot* OR intervention*) | 3,470 |
| S18 | (drink* OR alcohol*) N1 (drinking OR heavy OR risk* OR excess* OR consum* OR cessation OR cease OR ceasing OR quit* OR reduce* OR reducing OR reduction OR limit* OR free OR minimiz* OR minimis* OR decreas* OR lower* OR less OR lessen* OR "cut* back" OR "cut* down" OR change* OR changing OR improve* OR improving OR better OR behav* OR educat* OR prevent* OR intervention*) | 4,612 |
| S19 | (sunscreen OR suncream) N1 ("use of" OR using OR apply* OR application OR intervention* OR promot* OR educat*) | 34 |
| S20 | "sun protection" OR "sun exposure" OR "suncream use" OR "sunscreen use" OR "sun safety" OR "sun smart" OR sunsmart OR sunburn OR "sun damage" OR "UV radiation" OR "UV exposure" OR "UV protection" | 159 |
| S21 | "HPV vaccin*" OR "papillomavirus vaccin*" OR "Hep* B vaccin*" OR "HBV vaccin*" OR "HPV screening*" OR "papillomavirus screening*" | 168 |
| S22 | "cervical screening*" OR "vaginal smear*" OR "cervical smear*" OR mammogra* OR "breast screening*" OR "bowel screening*" OR "colorectal screening*" OR "CRC screening*" | 132 |
| S23 | S6 OR S7 OR S8 OR S9 OR S10 | 573,260 |
| S24 | S11 OR S12 OR S13 OR S14 OR S15 OR S16 OR S17 OR S18 OR S19 OR S20 OR S21 OR S22 | 74,255 |
| S25 | S5 AND S23 AND S24 | 39 |
| S26 | S4 OR S25 | 51 |

Medline eSign final search ran 27.09.24

|  | Searches | Results |
| --- | --- | --- |
| 1 | exp Electronic Health Records/ or Patient Portals/ or exp Medical Records Systems, Computerized/ or exp Health Records, Personal/ or *Medical Record Linkage/ or *Medical Records, Problem-Oriented/ | 54551 |
| 2 | Text Messaging/ or Electronic Mail/ or *Telephone/ or "Referral and Consultation"/ or Health Communication/ or Telemedicine/ or Digital Health/ | 133705 |
| 3 | "Early Detection of Cancer"/ or exp Health Behavior/ or Health Education/ or Health Promotion/ or Smoking Cessation/ or Smoking Reduction/ or Smoking Prevention/ or "Tobacco Use"/ or Tobacco Smoking/ or Cigarette Smoking/ or Overweight/ or Obesity/ or Obesity Management/ or Weight Reduction Programs/ or Diet, Reducing/ or Diet, Healthy/ or Healthy Lifestyle/ or Exercise/ or Alcohol Drinking/ or *Sunscreening Agents/ or *Sunburn/ or Papillomavirus Vaccines/ or Hepatitis B Vaccines/ or Vaginal Smears/ or Mammography/ or Cancer Vaccines/ | 1056480 |
| 4 | 1 and 2 and 3 | 372 |
| 5 | ("electronic health* record*" or "patient* portal*" or "web portal*" or "e-portal*" or eportal* or "electronic portal*" or "medical record* system*" or "personal health record*" or (patient* adj2 portal*) or "electronic medical record*" or "electronic patient* record*" or "computer* patient* record*" or "electronic patient* medical record*" or "digital health record*" or "digital patient* record*" or "computer* medical record*" or "digital medical record*" or "digital patient* medical record*" or "computer* health record*" or "computer* patient* medical record*").mp. | 103390 |
| 6 | (signpost* or "e-refer*" or erefer* or "text messag*" or "short message service*" or "multimedia messaging service*" or "rich communication service*" or "instant messag*" or "push notification*" or "app notification*" or "app messag*" or email* or "e-mail*" or "electronic mail*" or "electronic-mail*" or eHealth or "e-Health" or mHealth or "m-Health" or "mobile-health" or "one-click" or "opt-out").ti,ab. | 54809 |
| 7 | ((digital* or electronic* or mobile* or "mobile phone*" or "cell phone*" or telephone* or phone* or smartphone* or "smart phone*" or smartwatch* or "smart watch*" or automat* or virtual or video* or online or internet or computer* or remote or "provider-initiated" or proactive*) adj2 (messag* or refer* or screen* or reminder* or promot* or communicat* or text* or intervention* or appointment* or apps or applications)).ti,ab. | 83974 |
| 8 | (SMS adj4 (text* or messag* or reminder* or intervention* or educat* or appointment* or refer* or promot* or communicat*)).ti,ab. | 2579 |
| 9 | (MMS adj4 (text* or messag* or reminder* or intervention* or educat* or appointment* or refer* or promot* or communicat*)).ti,ab. | 170 |
| 10 | (RCS adj4 (text* or messag* or reminder* or intervention* or educat* or appointment* or refer* or promot* or communicat*)).ti,ab. | 129 |
| 11 | (health adj2 (behavior* or behaviour* or promot* or educat* or intervention*)).ti,ab. | 216837 |
| 12 | ((behavior or behaviour) adj2 (change* or changing or promot* or educat* or intervention*)).ti,ab. | 44715 |
| 13 | (cancer* adj2 (risk* or prevent* or screen*)).ti,ab. | 165398 |
| 14 | ((smoking or smoker* or tobacco or cigarette* or "nicotine replacement*") adj2 (cessation or cease or ceasing or quit* or prevent* or relapse or behav* or educat* or treatment* or therapy or therapies or intervention*)).ti,ab. | 63578 |
| 15 | ((obese or obesity or BMI or weight or overweight) adj2 (prevent* or educat* or risk* or reduce* or reducing or reduction or minimiz* or minimis* or decreas* or lower* or less* or change* or changing or improve* or improving or better or gain or loss* or lose or losing or excess* or intervention*)).ti,ab. | 320141 |
| 16 | ((diet* or eat*) adj2 (health* or behav* or change* or changing or improve* or improving or better or educat* or promot* or intervention*)).ti,ab. | 96619 |
| 17 | (("physical activity" or exercise) adj2 (health* or behav* or change* or changing or improve* or improving or better or educat* or promot* or intervention*)).ti,ab. | 74425 |
| 18 | ((drink* or alcohol*) adj2 (drinking or heavy or risk* or excess* or consum* or cessation or cease or ceasing or quit* or reduce* or reducing or reduction or limit* or free or minimiz* or minimis* or decreas* or lower* or less or lessen* or "cut* back" or "cut* down" or change* or changing or improve* or improving or better or behav* or educat* or prevent* or intervention*)).ti,ab. | 209674 |
| 19 | ((sunscreen or suncream) adj2 ("use of" or using or apply* or application or intervention* or promot* or educat*)).ti,ab. | 1795 |
| 20 | ("sun protection" or "sun exposure" or "suncream use" or "sunscreen use" or "sun safety" or "sun smart" or sunsmart or sunburn or "sun damage" or "UV radiation" or "UV exposure" or "UV protection").ti,ab. | 26834 |
| 21 | ("HPV vaccin*" or "papillomavirus vaccin*" or "Hep* B vaccin*" or "HBV vaccin*" or "HPV screening*" or "papillomavirus screening*").ti,ab. | 23010 |
| 22 | ("cervical screening*" or "vaginal smear*" or "cervical smear*" or mammogra* or "breast screening*" or "bowel screening*" or "colorectal screening*" or "CRC screening*").ti,ab. | 53675 |
| 23 | 6 or 7 or 8 or 9 or 10 | 130295 |
| 24 | 11 or 12 or 13 or 14 or 15 or 16 or 17 or 18 or 19 or 20 or 21 or 22 | 1159046 |
| 25 | 5 and 23 and 24 | 696 |
| 26 | 4 or 25 | 1028 |

PsycInfo eSign final search ran 27.09.24

| **#** | **Query** | **Results** |
| --- | --- | --- |
| S1 | DE "Electronic Health Records" OR DE "Medical Records" | 7,502 |
| S2 | DE "Text Messaging" OR DE "Computer Mediated Communication" OR DE "Telephone Systems" OR DE "Electronic Health Services" OR DE "Telemedicine" | 23,767 |
| S3 | (DE "Human Papillomavirus" OR DE "Hepatitis") AND (DE "Immunization" OR DE "Vaccination Attitudes") | 1,459 |
| S4 | DE "Cancer Screening" OR DE "Health Behavior" OR DE "Health Education" OR DE "Health Promotion" OR DE "Smoking Cessation" OR DE "Smoking Prevention" OR DE "Tobacco Smoking" OR DE "Overweight" OR DE "Obesity" OR DE "Weight Control" OR DE "Healthy Eating" OR DE "Lifestyle Changes" OR DE "Exercise" OR DE "Alcohol Use" OR DE "Tanning" OR DE "Mammography" | 225,080 |
| S5 | S3 OR S4 | 226,213 |
| S6 | S1 AND S2 AND S5 | 29 |
| S7 | TX ("electronic health* record*" OR "patient* portal*" OR "web portal*" OR "e-portal*" OR eportal* OR "electronic portal*" OR "medical record* system*" OR "personal health record*" OR (patient* N1 portal*) OR "electronic medical record*" OR "electronic patient* record*" OR "computer* patient* record*" OR "electronic patient* medical record*" OR "digital health record*" OR "digital patient* record*" OR "computer* medical record*" OR "digital medical record*" OR "digital patient* medical record*" OR "computer* health record*" OR "computer* patient* medical record*") | 8,228 |
| S8 | signpost* OR "e-refer*" OR erefer* OR "text messag*" OR "short message service*" OR "multimedia messaging service*" OR "rich communication service*" OR "instant messag*" OR "push notification*" OR "app notification*" OR "app messag*" OR email* OR "e-mail*" OR "electronic mail*" OR "electronic-mail*" OR eHealth OR "e-Health" OR mHealth OR "m-Health" OR "mobile-health" OR "one-click" OR "opt-out" | 33,971 |
| S9 | (digital* OR electronic* OR mobile* OR "mobile phone*" OR "cell phone*" OR telephone* OR phone* OR smartphone* OR "smart phone*" OR smartwatch* OR "smart watch*" OR automat* OR virtual OR video* OR online OR internet OR computer* OR remote OR "provider-initiated" OR proactive*) N1 (messag* OR refer* OR screen* OR reminder* OR promot* OR communicat* OR text* OR intervention* OR appointment* OR apps OR applications) | 61,712 |
| S10 | SMS N3 (text* OR messag* OR reminder* OR intervention* OR educat* OR appointment* OR refer* OR promot* OR communicat*) | 826 |
| S11 | MMS N3 (text* OR messag* OR reminder* OR intervention* OR educat* OR appointment* OR refer* OR promot* OR communicat*) | 38 |
| S12 | RCS N3 (text* OR messag* OR reminder* OR intervention* OR educat* OR appointment* OR refer* OR promot* OR communicat*) | 38 |
| S13 | health N1 (behavior* OR behaviour* OR promot* OR educat* OR intervention*) | 211,563 |
| S14 | (behavior OR behaviour) N1 (change* OR changing OR promot* OR educat* OR intervention*) | 58,437 |
| S15 | cancer* N1 (risk* OR prevent* OR screen*) | 14,390 |
| S16 | (smoking OR smoker* OR tobacco OR cigarette* OR "nicotine replacement*") N1 (cessation OR cease OR ceasing OR quit* OR prevent* OR relapse OR behav* OR educat* OR treatment* OR therapy OR therapies OR intervention*) | 30,796 |
| S17 | (obese OR obesity OR BMI OR weight OR overweight) N1 (prevent* OR educat* OR risk* OR reduce* OR reducing OR reduction OR minimiz* OR minimis* OR decreas* OR lower* OR less* OR change* OR changing OR improve* OR improving OR better OR gain OR loss* OR lose OR losing OR excess* OR intervention*) | 44,761 |
| S18 | (diet* OR eat*) N1 (health* OR behav* OR change* OR changing OR improve* OR improving OR better OR educat* OR promot* OR intervention*) | 39,250 |
| S19 | ("physical activity" OR exercise) N1 (health* OR behav* OR change* OR changing OR improve* OR improving OR better OR educat* OR promot* OR intervention*) | 28,433 |
| S20 | (drink* OR alcohol*) N1 (drinking OR heavy OR risk* OR excess* OR consum* OR cessation OR cease OR ceasing OR quit* OR reduce* OR reducing OR reduction OR limit* OR free OR minimiz* OR minimis* OR decreas* OR lower* OR less OR lessen* OR "cut* back" OR "cut* down" OR change* OR changing OR improve* OR improving OR better OR behav* OR educat* OR prevent* OR intervention*) | 72,340 |
| S21 | (sunscreen OR suncream) N1 ("use of" OR using OR apply* OR application OR intervention* OR promot* OR educat*) | 296 |
| S22 | "sun protection" OR "sun exposure" OR "suncream use" OR "sunscreen use" OR "sun safety" OR "sun smart" OR sunsmart OR sunburn OR "sun damage" OR "UV radiation" OR "UV exposure" OR "UV protection" | 1,176 |
| S23 | "HPV vaccin*" OR "papillomavirus vaccin*" OR "Hep* B vaccin*" OR "HBV vaccin*" OR "HPV screening*" OR "papillomavirus screening*" | 2,039 |
| S24 | "cervical screening*" OR "vaginal smear*" OR "cervical smear*" OR mammogra* OR "breast screening*" OR "bowel screening*" OR "colorectal screening*" OR "CRC screening*" | 4,657 |
| S25 | S8 OR S9 OR S10 OR S11 OR S12 | 86,300 |
| S26 | S13 OR S14 OR S15 OR S16 OR S17 OR S18 OR S19 OR S20 OR S21 OR S22 OR S23 OR S24 | 412,887 |
| S27 | S7 AND S25 AND S26 | 317 |
| S28 | S6 OR S27 | 328 |

Scopus eSign final search ran 27.09.24

**1,494 documents found**

( TITLE-ABS-KEY ( "electronic health* record*" OR "patient* portal*" OR "web portal*" OR "e-portal*" OR eportal* OR "electronic portal*" OR "medical record* system*" OR "personal health record*" OR ( patient* W/1 portal* ) OR "electronic medical record*" OR "electronic patient* record*" OR "computer* patient* record*" OR "electronic patient* medical record*" OR "digital health record*" OR "digital patient* record*" OR "computer* medical record*" OR "digital medical record*" OR "digital patient* medical record*" OR "computer* health record*" OR "computer* patient* medical record*" ) ) AND ( ( TITLE-ABS-KEY ( signpost* OR "e-refer*" OR erefer* OR "text messag*" OR "short message service*" OR "multimedia messaging service*" OR "rich communication service*" OR "instant messag*" OR "push notification*" OR "app notification*" OR "app messag*" OR email* OR “e-mail*” OR "electronic mail*" OR "electronic-mail*" OR ehealth OR "e-Health" OR mhealth OR "m-Health" OR "mobile-health" OR "one-click" OR "opt-out" ) ) OR ( TITLE-ABS-KEY ( ( digital* OR electronic* OR mobile* OR "mobile phone*" OR "cell phone*" OR telephone* OR phone* OR smartphone* OR "smart phone*" OR smartwatch* OR "smart watch*" OR automat* OR virtual OR video* OR online OR internet OR computer* OR remote OR "provider-initiated" OR proactive* ) W/1 ( messag* OR refer* OR screen* OR reminder* OR promot* OR communicat* OR text* OR intervention* OR appointment* OR apps OR applications ) ) ) OR ( TITLE-ABS-KEY ( sms W/3 ( text* OR messag* OR reminder* OR intervention* OR educat* OR appointment* OR refer* OR promot* OR communicat* ) ) ) OR ( TITLE-ABS-KEY ( mms W/3 ( text* OR messag* OR reminder* OR intervention* OR educat* OR appointment* OR refer* OR promot* OR communicat* ) ) ) OR ( TITLE-ABS-KEY ( rcs W/3 ( text* OR messag* OR reminder* OR intervention* OR educat* OR appointment* OR refer* OR promot* OR communicat* ) ) ) ) AND ( ( TITLE-ABS-KEY ( health W/1 ( behavior* OR behaviour* OR promot* OR educat* OR intervention* ) ) ) OR ( TITLE-ABS-KEY ( ( behavior OR behaviour ) W/1 ( change* OR changing OR promot* OR educat* OR intervention* ) ) ) OR ( TITLE-ABS-KEY ( cancer* W/1 ( risk* OR prevent* OR screen* ) ) ) OR ( TITLE-ABS-KEY ( ( smoking OR smoker* OR tobacco OR cigarette* OR "nicotine replacement*" ) W/1 ( cessation OR cease OR ceasing OR quit* OR prevent* OR relapse OR behav* OR educat* OR treatment* OR therapy OR therapies OR intervention* ) ) ) OR ( TITLE-ABS-KEY ( ( obese OR obesity OR bmi OR weight OR overweight ) W/1 ( prevent* OR educat* OR risk* OR reduce* OR reducing OR reduction OR minimiz* OR minimis* OR decreas* OR lower* OR less* OR change* OR changing OR improve* OR improving OR better OR gain OR loss* OR lose OR losing OR excess* OR intervention* ) ) ) OR ( TITLE-ABS-KEY ( ( diet* OR eat* ) W/1 ( health* OR behav* OR change* OR changing OR improve* OR improving OR better OR educat* OR promot* OR intervention* ) ) ) OR ( TITLE-ABS-KEY ( ( "physical activity" OR exercise ) W/1 ( health* OR behav* OR change* OR changing OR improve* OR improving OR better OR educat* OR promot* OR intervention* ) ) ) OR ( TITLE-ABS-KEY ( ( drink* OR alcohol* ) W/1 ( drinking OR heavy OR risk* OR excess* OR consum* OR cessation OR cease OR ceasing OR quit* OR reduce* OR reducing OR reduction OR limit* OR free OR minimiz* OR minimis* OR decreas* OR lower* OR less OR lessen* OR "cut* back" OR "cut* down" OR change* OR changing OR improve* OR improving OR better OR behav* OR educat* OR prevent* OR intervention* ) ) ) OR ( TITLE-ABS-KEY ( ( sunscreen OR suncream ) W/1 ( "use of" OR using OR apply* OR application OR intervention* OR promot* OR educat* ) ) ) OR ( TITLE-ABS-KEY ( "sun protection" OR "sun exposure" OR "suncream use" OR "sunscreen use" OR "sun safety" OR "sun smart" OR sunsmart OR sunburn OR "sun damage" OR "UV radiation" OR "UV exposure" OR "UV protection" ) ) OR ( TITLE-ABS-KEY ( "HPV vaccin*" OR "papillomavirus vaccin*" OR "Hep* B vaccin*" OR "HBV vaccin*" OR "HPV screening*" OR "papillomavirus screening*" ) ) OR ( TITLE-ABS-KEY ( "cervical screening*" OR "vaginal smear*" OR "cervical smear*" OR mammogra* OR "breast screening*" OR "bowel screening*" OR "colorectal screening*" OR "CRC screening*" ) ) )
