# Supplementary material for: Implementation of electronic signposting to interventions that prevent cancer: A realist review
Source: PLOS Digit Health. 2026 Oct 5;5(10):e0001770. doi: 10.1371/journal.pdig.0001770 (PMC13637878; doi:10.1371/journal.pdig.0001770)
Supplement: Supplementary file 3 [file pdig.0001770.s004.docx]

**S4 Table: Refined programme theory of electronic signposting to cancer prevention interventions**

| **Refined CMOCs (context-mechanism-outcome configurations)** | **Example excerpts** |
| --- | --- |
| ***Patient ‘buy-in’ to eSignposting*** *(how motivated and encouraged patients are to engage with eSignposting messages, or not)* |  |
| CMOC 1  If patient lifestyle behaviours (C) are well matched to the eSignposting characteristics because the messages specify an achievable gain-framed goal, then patients will be motivated (M) to engage with eSignposting messages (O) | Ackerman 2023  ‘Stakeholders cautioned that encouragement of a weight loss goal in a MyChart message could be perceived as inappropriate by some patients; they advised framing the goal and offering resources as means to improve cardiovascular risk factor control. They also advised recommending a relatively simple initial goal of “about 10 pounds over 10 weeks,” believing this would be acceptable, appropriate, and achievable for most patients.’ |
| CMOC 2  If patient lifestyle behaviours (C) are well matched to the eSignposting characteristics because the messages are salient in wording and design, then patients will be motivated (M) to engage with eSignposting messages (O) | Azevedo 2023  ‘We encourage developers to consider our findings to assist successful use of digital health solutions by including designs with color-coded number lines, emojis, the use of conversational agents (CAs), gamification, as well as other features and mechanisms that promote appropriate affective response and gist memory.’ |
| CMOC 3  If patient lifestyle behaviours (C) are well matched to the eSignposting characteristics because the messages fit with social norms, then patients will be motivated (M) to engage with eSignposting messages (O) | Mehta 2018  ‘Society expects individuals to respond to cooperative and altruistic actions with a similar action.’ |
| CMOC 4  If patient cultural practices (C) are well matched to the eSignposting characteristics, because the messages are sensitive to native language, history and cultural beliefs, then patients will be empowered (M) to engage with eSignposting messages (O) | Bayard 2022  ‘EHRs [electronic health records] are primarily in English, which disenfranchises patients whose native language is not English.’  ‘Mistrust and privacy are also important factors. Patients with low socioeconomic status and of racial and ethnic minority groups may feel mistrust due to historic mistreatment of vulnerable populations and privacy, which may contribute to disparities in enrolment.’ |
| CMOC 5  If patient beliefs are well matched to the eSignposting characteristics, because the messages are autonomy supporting, then patients will be committed (M) to engage with eSignposting messages (O) | Lafata 2019  ‘We use principles from Self-Determination Theory to guide the tone of the written messages and ensure they are autonomy supporting and are not overly directive or controlling.’ |
| CMOC 6  If patient comprehension (C) is well matched to the eSignposting characteristics because the instructions are informative and delivered in clear and simple terms, then patients will feel informed (M) to engage with eSignposting messages (O) | Thompson 2019  ‘Emphasized the importance of providers relaying colon cancer information in “clear and simple terms” that they could understand and share with their family.’ |
| CMOC 7  If patient emotional state (C) is well matched to the eSignposting characteristics, because the messages are sensitive to emotional wellbeing, then patients will be encouraged (M) to engage with eSignposting messages (O) | Lockwood 2023  ‘Individuals with very high BMI tend to delay or avoid healthcare visits and tend to have lower participation in lifestyle change programs, in part due to emotional barriers, such as perceived weight stigma from providers.’ |
| CMOC 8  If patient financial circumstances (C) are well matched to accessing eSignposting messages and the service which is being signposted to, and costs/incentives are made explicit, then patients will be enabled (M) to engage with eSignposting messages (O) | Muller 2017  ‘Services were offered without requiring out-of-pocket payments. This factor removed insurance and other financial barriers to CRC [colorectal cancer] screening’ |
| CMOC 9  If patient physical functioning (C) is well matched to accessing eSignposting messages and the service which is being signposted to, then patients will be enabled (M) to engage with eSignposting messages (O) | Khadjesari 2022 (appendix 1)  ‘Disabilities were raised as a potential issue e.g. mental illness, sight issues, manual dexterity. Suggestion to ensure the link was clear on the message.’ |
| CMOC 10  If patient lifestyle circumstances (C) are well matched to the eSignposting message and service characteristics because these offer flexibility and convenience, then patients will be enabled (M) to engage with eSignposting messages (O) | Erdmann 2022  ‘It is likely that the asynchronous care was more attractive to patients because they could obtain help and support directly from their PCP [primary care provider] (eg, prescription medication) without having to call, schedule an appointment, and take time to attend in-person care.’ |
| CMOC 11  If patient lifestyle circumstances (C) are well matched to the time of eSignposting message sending and there is flexibility in the time to access/ action messages, then patients will be encouraged (M) to engage with eSignposting messages (O) | Hess 2014  ‘By providing reminders through the PHR [personal health record], patients are able to choose when they receive the message (log-on to the PHR) and are able to take action regarding the message immediately by scheduling an appointment or sending a request to the clinician’s office to have a test or procedure ordered.’ |
| CMOC12  If patient lifestyle circumstances (C) are well matched to eSignposting message frequency, then patients will feel ‘nudged’ and not bombarded (M) and will engage with eSignposting messages (O) | Miller 2021  ‘Finding the optimal number and frequency of electronic notifications and reminders is a high priority of web-based approaches in order to sustain high participation.’ |
| ***Provider ‘buy-in’ to eSignposting*** *(how motivated and enabled different professionals within a healthcare setting are to deliver eSignposting messages, or not)* |  |
| CMOC 13  If organisational priorities (C) are well matched or can be focused on eSignposting aims, then staff will be committed (M) to deliver eSignposting messages (O) | McVay 2022  ‘Interviews with enrolled PCPs [primary care providers] suggest that success in recruiting PCPs may be attributable to the PCPs' overall interest in connecting patients to weight management resources’ |
| CMOC 14  If decision-makers (C) accept and approve eSignposting strategies, then they will be committed (M) to deliver eSignposting messages (O) | Khadjesari 2022  ‘Senior-level leadership and buy-in from a public health consultant within the digital health team were instrumental in the successful implementation of this project.’ |
| CMOC 15  If personnel of different rank and file (C) perceive eSignposting to offer relative advantage, then they will be motivated (M) to deliver eSignposting messages (O) | Khadjesari 2022  ‘Advantages were seen as outweighing disadvantages’ |
| CMOC 16  If organisational incentives and targets (C) can be well matched with eSignposting aims, then staff will be incentivised (M) to deliver eSignposting messages (O) | Sadasivam 2011  ‘Pilot data immediately indicated that financial incentives would spur participation.’ |
| CMOC 17  If organisational finances (C) are well matched with eSignposting messages offering low comparative cost, and funding is sustained, then staff will be enabled (M) to deliver eSignposting messages (O) | Kearney 2024  ‘Using existing infrastructure, such as portal questionnaires and text surveys, is a low-cost, readily available way to asynchronously engage with patients and gather more structured data for clinical use.’ |
| CMOC 18  If organisations have political and commissioner support to use eSignposting (C), then staff will be enabled (M) to deliver eSignposting messages (O) | El-Osta 2021  ‘Commissioners and public health local area teams therefore need to work collaboratively to ensure a coordinated approach to using the quality standard to improve outcomes for people who want to stop smoking, including leveraging IT to streamline access to smoking cessation support. A progressive commissioning landscape by local authorities and public health local area teams that would enable the disbursement of activity-based funds to primary care led smoking cessation services is indicated.’ |
| CMOC 19  If staff are aware of the benefits of services patients are being signed to (C), then staff will be motivated (M) to deliver eSignposting messages (O) | Houston 2015 (table 1)  ‘Implementation facilitator…Perceived potential to affect care’ |
| ***Patient perceived system usability*** *(if patients access and confidence with technology enables them to engage with eSignposting messages, or not)* |  |
| CMOC 20  If patients have suitable digital access (C), and the eSignposting mode uses the most popular patient communication mode, then patients will be enabled (M) to engage with eSignposting messages (O) | Muller 2017  ‘Text messaging might be useful for addressing health disparities in populations who face barriers to accessing personal computers and the Internet but have high rates of mobile telephone use.’ |
| CMOC 21  If patients have suitable digital access (C), and the eSignposting mode and service use multiple channels then patients will be enabled (M) to engage with eSignposting messages (O) | Ackermann 2023  ‘We also considered how to offer the resulting services using an array of different channels (e.g., MyChart, telephone, smartphone application, and fitness facility) to maximize reach by appealing to different segments of the target population.’ |
| CMOC 22  If patients’ digital capabilities (C), are well matched with the eSignposting mode, because the messages are user-friendly, then patients will be enabled (M) to engage with eSignposting messages (O) | Chevinsky 2021  ‘To design the patient interface in a clear, friendly, and nonjudgemental fashion’ |
| CMOC 23  If patient lifestyle circumstances (C) are well matched to eSignposting mode, by ensuring messages are easy e.g. ‘one-click’, then patients will perceive eSignposting as ‘low-effort’ (M) and will engage with eSignposting messages (O) | Ackermann 2023 (additional file 1)  ‘Clickable link to a web page that provided simple summaries of the components and differences between each program with a single “enroll me” button that helped enroll the patient’ |
| CMOC 24  If patient neurological functioning (C) is well matched to the eSignposting characteristics, because the messages are brief, clear and simplistic, then patients will experience ‘low cognitive load’ (M) and be able to engage with eSignposting messages (O) | Stephens 2019  ‘Text messages are also useful across populations with various levels of literacy since each text message can only be 160 characters, which facilitates the use of a minimal number of short, simple words.’ |
| ***Provider perceived system usability*** *(if digital infrastructure enables professionals within a healthcare setting to deliver eSignposting messages, or not)* |  |
| CMOC 25  If organisation digital infrastructure is robust (C), and the eSignposting method can be integrated with systems, then staff will be enabled (M) to deliver eSignposting messages (O) | Yoon 2021 (personal communication from co-author)  ‘In a setting like Kaiser Permanente (integrated practice with robust EMR [electronic medical record]), I believe it would be successful; not necessarily so in other areas.’ |
| CMOC 26  If electronic health record and messaging systems are accurate (C), and eSignposting complements data accuracy, then staff will be enabled (M) to deliver eSignposting messages (O) | Begnaud 2017  ‘Future steps to improve access to LCS [lung cancer screening] for eligible persons may include EHR [electronic health record] system tools to document an accurate and detailed smoking history, which will permit systematic identification of possibly eligible persons.’ |
| CMOC 27  If electronic health record and messaging systems are user-friendly (C), and eSignposting is quick, simplistic and compatible with systems, then staff will be enabled (M) to deliver eSignposting messages (O) | Fischer 2013  ‘The EHR [electronic health record] allowed the person running the reports to send the reminder messages or letters to all of the participants on the reports directly from the screen showing the report results.’ |
| CMOC 28  If electronic health record and messaging systems are adaptable (C), and eSignposting is compatible with varied systems, then staff will be enabled (M) to deliver eSignposting messages (O) | Dharod 2019  ‘Query to identify potential screening candidates could also be revised to match a health system’s data architecture.’ |
| CMOC 29  If electronic health record and messaging systems are time and resource efficient e.g. fully automated/ AI generated (C), then staff will perceive message sending as ‘low-effort’ (M) and will be more likely to deliver eSignposting messages (O) | Lafata 2022  ‘By leveraging the functionality of an EHR [electronic health record], we were able to automate both the identification of patients and the delivery of an intervention designed to engage and support patients to complete CRC [colorectal cancer] screening after a physician recommended it. Doing so ensured the intervention’s integration with existing primary care workflows without requiring busy primary care providers and staff to alter current practices.’ |
| ***Patient ‘techno-optimism’ of eSignposting*** *(if patients are optimistic in the technology of eSignposting messages, or not)* |  |
| CMOC 30  If patient attitudes towards digital technology are positive (C), or positivity can be encouraged through favourable design, then patients will be optimistic (M) to engage with eSignposting messages (O) | Azevedo 2023  ‘Designers can improve users’ onboarding and initial experiences with digital health solutions, which is known to be related to technology adoption, perceived ease of use, and usefulness.’ |
| CMOC 31  If patient digital usage is frequent (C), or technical use can be supported by training and simple design, then patients will be confident (M) to engage with eSignposting messages (O) | Liang 2022  ‘Once patients learned how to schedule appointments through the patient portal, they used the portal to schedule appointments from that time onward.’ |
| CMOC 32  If patient perception of technology (C) is satisfied that eSignposting messages are authentic and trustworthy, then patients will feel reassured (M) to engage with eSignposting messages (O) | McIntosh 2025  ‘Many people who received the SMS felt that was considered trustworthy and reliable, so they took notice of it’ |
| CMOC 33  If patient perception of technology (C) is satisfied that eSignposting messages are private and secure, then patients will feel reassured (M) to engage with eSignposting messages (O) | McVay 2022  ‘They trusted that the information was secure and would stay private’ |
| ***Provider ‘techno-optimism’ of eSignposting*** *(if professionals within a healthcare setting are optimistic in the technology of delivering eSignposting messages, or not)* |  |
| CMOC 34  If data governance standards are met (C), then staff will be reassured (M) to deliver eSignposting messages (O) | Lockwood 2023  ‘Appropriate safeguards were taken to prevent any unauthorized use or disclosure of personal health information and to implement the administrative, physical, and technical safeguards to protect the confidentiality, integrity, and availability of protected health information.’ |
| CMOC 35  If organisation culture enables sufficient resourcing for digital systems (C) and eSignposting uses few resources, then staff will have greater capacity (M) to deliver eSignposting messages (O) | Muller 2017  ‘Another strength of the current study was that the intervention can be implemented and sustained, as designed, in the frequently overburdened and underfunded settings experienced by many tribal health care systems.’ |
| CMOC 36  If organisation culture enables sufficient training for digital systems (C) and eSignposting can be aligned with training needs, then staff will feel capable (M) to deliver eSignposting messages (O) | Ackermann (additional file)  ‘Clinicians and staff members recommended that health system leaders or the research team use existing practice meetings and also offer brief training sessions with key service professionals to ensure they understand any new responsibilities, use of new technology applications, and how their performance will be measured.’ |
| CMOC 37  If organisation culture enables sufficient flexibility for new digital systems to be introduced (C), and eSignposting can be equally flexible, then staff will be optimistic (M) to deliver eSignposting messages (O) | Stephens 2019  ‘Text message reminders are a flexible platform that can be used either for simple prompts or reminders that inform a patient or family when a vaccine is needed, or for more complex interactions including the embedding of educational information and interactive responses.’ |
| CMOC 38  If organisation culture enables sufficient flexibility for new digital systems to be introduced (C), and eSignposting minimises workflow disruption, then staff will be optimistic (M) to deliver eSignposting messages (O) | Houston 2015  ‘Each practice was allowed to consider how to best integrate the referrals into their workflow, allowing variation in implementation fidelity.’ |
| CMOC 39  If organisation culture enables sufficient flexibility for new digital systems to be introduced (C), and eSignposting is supported by an external party, then staff will be optimistic (M) to deliver eSignposting messages (O) | Houston 2015  ‘Created a proactive helpdesk to enhance our study personnel’s availability for technical assistance.’ |
| CMOC 40  If organisations have digital experts, or have digital champions (C) to promote eSignposting, then staff will perceive greater collective efficacy (M) to deliver eSignposting messages (O) | Houston 2015  ‘In our pilot test, staff turnover was identified as a challenge to sustained implementation, so each practice was asked to identify two implementation coordinators (physicians, nurses, or other staff). These two implementation coordinators acted as points of contact and champions. They were trained in the referral intervention and acted as train-the-trainers providing information to the rest of the practice and encouraging adoption.’ |
| CMOC 41  If organisations have gained previous digital experience (C), then staff will perceive greater collective efficacy (M) to deliver eSignposting messages (O) | Ackerman 2023 (additional file 1)  ‘Members of the health system’s technology support team determined technical feasibility and developed prototypes for testing and refinement.’ |
| CMOC 42  If organisations have no previous digital experience (C) and champion novel opportunities, then staff will be enthusiastic (M) to deliver eSignposting messages (O) | Yoon 2021  ‘Internal presentations and discussions of the data have led to an interest in expanding the process to automate other screenings’ |
| CMOC 43  If existing systems (C) enable feedback on eSignposting’s impact, then staff will be adaptive (M) and able to deliver eSignposting messages (O) | Houston 2015  ‘Positive feedback to practices—practices can track registration and cessation progress of their patients and send individual doctor-to-patient secure messages to encourage participation.’ |
| CMOC 44  If existing systems (C) enable integration of eSignposting with other services, then staff will be adaptive (M) and able to deliver eSignposting messages (O) | Bayard 2022  ‘EHRs [electronic health records] can also centralize provider communication that is fragmented across multiple institutions, which often leads to worse outcomes for patients.’ |
| ***Patient-professional solidarity*** *(how complementary eSignposting is with patient-professional relationships, or not)* |  |
| CMOC 45  If patient-provider norms (C) are complementary to eSignposting because messages are designed to supplement current healthcare practices, then patient-provider relationships will be empowered (M) and eSignposting will have greater acceptability to patients and providers (O) | Begnaud 2017  ‘Such systematic identification should not supplant shared decision making, but, rather, should be a tool to improve the timely screening for eligible and interested persons.’ |
| CMOC 46  If patient-provider norms (C) are complementary to eSignposting because messages are designed to provide added equality-centred patient benefit and reduce inequalities, then patient-provider relationships will be empowered (M) and eSignposting will have greater acceptability to patients and providers (O) | Peitzmeier 2016  ‘Effective outreach programs that engage underscreened populations are clearly needed.’ |
| CMOC 47  If patient-provider relationships (C) are maintained by tailoring eSignposting messages to patient needs and preferences, then patients and providers will be empowered (M) and eSignposting will have greater acceptability to patients and providers (O) | Combest 2019  ‘Studies should also assess participants’ readiness to change their lifestyle behaviors, so that messaging is tailored based on stage of readiness to change.’ |
| CMOC 48  If patient-provider relationships (C) are maintained by personalising eSignposting messages and privacy of name concerns are addressed, then patients will perceive greater relevance (M) and eSignposting will have greater acceptability to patients and providers (O) | Weaver 2015  ‘A distinct advantage of text messages is the ability to easily tailor them to the individual. Our results reveal some tailoring may be required to craft text messages that are broadly appealing, but this should be achievable with relatively simple algorithms (eg, patient preference for first or last name).’ |
| CMOC 49  If patient ethics, including adding an opt-out of data use option (C), are met by eSignposting messages, then patients and providers will be less resistant to eSignposting messages (M) and eSignposting will have greater acceptability to patients and providers (O) | Bayard 2022  ‘Portal enrollment could be restructured to allow universal access and “opt-out” policies, which would eliminate requirements for active enrollment.’ |
| CMOC 50  If patient eligibility e.g. BMI cut-off, is suitable (C) and can be well matched to eSignposting message content, then patients will perceive greater relevance (M) and eSignposting will have greater acceptability to patients and providers (O) | Ackermann 2023 (additional file)  ‘Stakeholders also believed that targeting patients with mildly overweight BMI’s increased the possibility that weight loss might not be appropriate or acceptable to some patients; they recommended targeting those with BMI ≥27 kg/m2.’ |
| CMOC 51  If patients and providers are aware that eSignposting messages will be sent and are aware of their content (C), then they will be better prepared for the messages and the intervention (M) and eSignposting will have greater acceptability to patients and providers (O) | Combest 2019  ‘Increased email communications may cause this health promotion message to be considered among “junk” or “spam” email and automatically filtered. Simple instructions given by providers and administrative staff could help extend its usage and alleviate these barriers.’ |
| CMOC 52  If diverse patients and providers have contributed to the design of eSignposting message mode and content (C), then patients and providers will be empowered (M) and eSignposting will have greater acceptability to patients and providers (O) | Eden 2020  ‘The development process included interviews with patients and clinical experts to assess face validity, patient comprehension, and ease of use.’ |
| CMOC 53  If eSignposting messages and the services being signposted to are evidence-based (C), then patients and providers will be more engaged in the intervention (M) and eSignposting will have greater acceptability to patients and providers (O) | Khadjesari 2022  ‘The apps are theoretically informed and use evidence-based behavior change techniques, such as goal setting and self-monitoring.’ |
| CMOC 54  If eSignposting messages and the services being signposted to are endorsed by providers (C), then patients will feel reassured to engage with eSignposting messages (M) and eSignposting will have greater acceptability to patients and providers (O) | Wood 2022  ‘There is strong evidence to suggest that GP endorsement of screening can increase participation’ |
| CMOC 55  If eSignposting is linked to a patient appointment or within a short timeframe of this (C), then patients and providers will be motivated (M) and eSignposting will have greater acceptability to patients and providers (O) | Combest 2019  ‘The health messaging sent before a nutrition appointment, for example, could be used to create anticipation or for dialogue during the appointment. Furthermore, an email message after the nutrition appointment could be used to maintain momentum towards reaching nutrition goals.’ |
| CMOC 56  If eSignposting is not linked to a patient appointment (C), then patients and providers will experience less burden (M) and eSignposting will have greater acceptability to patients and providers (O) | Rief 2017  ‘Extended the patient–provider relationship beyond the bounds of increasingly time-constrained office visits and cultivated opportunities for active involvement by participants in their own care.’ |
| CMOC 57  If eSignposting target behaviours are presented non-judgmentally, during or outside of patient appointments (C), then patients and providers will not be concerned about stigma (M) and eSignposting will have greater acceptability to patients and providers (O) | Mahoney 2018  ‘An AVR [automated voice recognition] system may better address tobacco dependence given the hesitancy of many health care professionals and individual patients toward discussing this information.’ |
